# Seven Novel Genes Related to Cell Proliferation and Migration of VHL-Mutated Pheochromocytoma

**DOI:** 10.3389/fendo.2021.598656

**Published:** 2021-03-22

**Authors:** Shuai Gao, Longfei Liu, Zhuolin Li, Yingxian Pang, Jiaqi Shi, Feizhou Zhu

**Affiliations:** ^1^Department of Biochemistry and Molecular Biology, School of Life Sciences, Central South University, Changsha, China; ^2^Department of Urology, Xiangya Hospital, Central South University, Changsha, China; ^3^Hunan Key Laboratory of Animal Models for Human Diseases, Central South University, Changsha, China

**Keywords:** pheochromocytoma, Von Hippel-Lindau (VHL), connective tissue growth factor (CTGF), syndecan binding protein (SDCBP), cellular communication network factor 1 (CCN1), collagen type III alpha 1 chain (COL3A1), collagen type V alpha 2 chain (COL5A2), serpin family E member 1 (SERPINE1)

## Abstract

Pheochromocytoma, as a neuroendocrine tumor with the highest genetic correlation in all types of tumors, has attracted extensive attention. Von Hipper Lindau (VHL) has the highest mutation frequency among the genes associated with pheochromocytoma. However, the effect of VHL on the proteome of pheochromocytoma remains to be explored. In this study, the VHL knockdown (VHL-KD) PC12 cell model was established by RNA interference (shRNA). We compared the proteomics of VHL-KD and VHL-WT PC12 cell lines. The results showed that the expression of 434 proteins (VHL shRNA/WT > 1.3) changed significantly in VHL-KD-PC12 cells. Among the 434 kinds of proteins, 83 were involved in cell proliferation, cell cycle and cell migration, and so on. More importantly, among these proteins, we found seven novel key genes, including Connective Tissue Growth Factor (CTGF), Syndecan Binding Protein (SDCBP), Cysteine Rich Protein 61 (CYR61/CCN1), Collagen Type III Alpha 1 Chain (COL3A1), Collagen Type I Alpha 1 Chain (COL1A1), Collagen Type V Alpha 2 Chain (COL5A2), and Serpin Family E Member 1 (SERPINE1), were overexpressed and simultaneously regulated cell proliferation and migration in VHL-KD PC12 cells. Furthermore, the abnormal accumulation of HIF2α caused by VHL-KD significantly increased the expression of these seven genes during hypoxia. Moreover, cell-counting, scratch, and transwell assays demonstrated that VHL-KD could promote cell proliferation and migration, and changed cell morphology. These findings indicated that inhibition of VHL expression could promote the development of pheochromocytoma by activating the expression of cell proliferation and migration associated genes.

## Introduction

Pheochromocytoma and paraganglioma (PPGL) are rare neuroendocrine tumors with a incidence of about six patients per million, of which approximately 10% of patients are diagnosed as malignant tumors (1, 2). Although the malignancy rate of PPGL was not high, but it had relatively high genetic relevance and germline mutation rate (3). According to the statistics, about 40% of PPGL were related to germline mutations (4, 5). So far, about 20 susceptibility genes were found in PPGL, which could be divided into three different clusters: the Cluster 1, which was related to the pseudo-hypoxia signaling pathway, including VHL, SDHx, FH, PHD2, and HIF2α, which could be divided into two subgroups: the tricarboxylic acid (TCA) cycle and the VHL/HIF axis (6–8); the Cluster 2, which was related to kinase signaling pathways including RET, NF1, MAX, KIF1B, H-RAS, and TMEM127; and the Cluster 3 was related to the Wnt signaling pathway, including CSDE1 and MAML3 fusion gene (5, 9–13).

Among the PPGL susceptibility genes, the genes involved in the VHL/HIF axis had the highest mutation frequency (14, 15). The pVHL was a component of the ubiquitin ligase complex, where VHL recognized the substrate. In addition to pVHL, the ubiquitin ligase complex also contained elongin B, elongin C, Cul2, and Rbx1 (16). Moreover, pVHL played an important role in the formation of extracellular matrix and epithelial differentiation (17), and was necessary for the interaction between epithelial cells and extracellular matrix (18). The abnormal function of pVHL prevented the ubiquitin ligase complex from ubiquitating the α-subunit of the heterodimeric transcription factor HIF (hypoxia-inducible factor), thereby preventing the degradation of HIFα in the proteasome (19, 20). Some studies reviewed the molecular functions of pVHL, indicating that pVHL could regulate many processes such as cell cycle (21), mRNA stability, and hypoxia induced gene expression (16, 22). Hypoxia inducible-factor 2α (HIF2α) inhibition was necessary for pVHL to inhibit tumor (23). Meanwhile, there were three kinds of HIFα proteins in human, including hypoxia-inducible factor 1α (HIF1α), HIF2α, and hypoxia-inducible factor 3α (HIF3α) (24), in which mutations in HIF2α or downstream pseudo-hypoxia signaling pathway–related genes were susceptible to PPGL (25).

Moreover, HIF2α mutations were found in a new unique type of syndrome. The new syndrome disease carrying PPGL-somatostatinoma-polycythemia greatly promoted our understanding of the key molecular mechanisms of PPGL (26, 27). Melanie J et al. reported that a gain-of-function missense mutation occurred in HIF2α and impaired the hydroxylation of HIF2α protein. This mutation not only maintained the stable conformation of HIF2α, but also caused the onset of polycythemia (28). More gain-of-function missense mutations in HIF2α were found in PPGL associated with polycythemia (29). HIF2α was also considered to be a key regulator of erythropoiesis (30). However, many researchers successively reported that HIF2α mutations were found in PPGL patients with or without polycythemia and somatostatinoma (27, 31–35). The patients’ multiple organ involvement and distant metastasis indicated that the HIF2α mutation occurred in the early life or embryonic development stage. And the different periods of HIF2α somatic mutations in pregnancy might affect the later phenotype of the syndrome (36). At the same time, Lorenzo et al. described a new germline mutation of HIF2α in PGLs patients (37). On the other hand, HIF2α was necessary for the synthesis of catecholamines. The analysis of the patient’s clinical manifestations and HIF2α imbalance reflected the role of HIF2α in preferential norepinephrine synthesis. So HIF2α mutation might provide more guidance for the molecular typing and prognosis of PPGL (38, 39). Furthermore, studies showed that overexpression of HIF2α might lead to the formation of diffuse clusters and the appearance of pseudopodia (40), and enhance cell proliferation and metastatic load (25, 41). Therefore, HIF2α played an important role in pheochromocytoma. The discovery of new downstream genes driven by HIF2α might provide new ideas for the treatment of pheochromocytoma.

HIF2α stabilization was also found in patients with VHL mutation (42). There were many reports on the function of pVHL, and it was reported that pVHL could cause abnormal accumulation of HIF2α thereby activating the expression of downstream target genes of HIF2α. In this study, we used the latest high-sensitivity proteomics platform of isobaric labels tandem mass tags (TMT) to discover the novel proteins involved in pVHL/HIF2α axis. This will help us to understand the pathogenic mechanism of VHL dysfunction, guide the treatment of clinical diseases and optimize the prognosis.

## Materials and Methods

### Tumor Tissue Samples

From December 2017 to June 2018, 30 cases of tumor tissue and para-carcinoma medulla samples were collected from PPGL patients undergoing laparoscopic surgery in Xiangya Hospital, Hunan, China. Through imaging examinations (computed tomography and color Doppler ultrasound) and laboratory examinations, all patients were diagnosed as PPGL. All PPGL samples confirmed by pathological examination were stored at −80°C for later use. Subsequently, Sanger and next-generation sequencing confirmed VHL mutations in four patients who had not received any treatment before surgery. All patients signed an informed consent form before surgery and the study was approved by the Ethical Committee of the School of Life Sciences, Central South University.

### Cell Culture and Proliferation Assay

The rat adrenal pheochromocytoma PC12 cell line was obtained from the Advanced Research Center of Central South University (Changsha, China). The cells were transported on dry ice, and after receiving, the cells were resuscitated according to standard methods, sub-cultured, and cryopreserved (43, 44). The frozen cells were stored in liquid nitrogen and the recovered PC12 cells were routinely inoculated into RPMI-1640 medium (GIBCO, Carlsbad, CA, USA) containing 10% fetal bovine serum (FBS) (GIBCO, Carlsbad, CA, USA), penicillin (100 U/ml), and streptomycin (100 μg/ml). The cells were maintained in humidified incubator containing 5% CO2 at 37°C for routine culture. In order to analyze the proliferation of the cells, the cells were seeded in six-well plates with 1 × 10^5^ cells per well. The cells were trypsinized every other day for 3 consecutive days and counted with CellDrop BF counter (Denovix). To simulate a hypoxic environment, different types of PC12 cells were cultured in a hypoxic incubator containing 94% N_2_, 5% CO_2_, and 1% O_2_ for 24 h or 21% O_2_, and then the downstream experiments were performed immediately.

### VHL RNAi

According to VHL target gene sequence and RNAi sequence design principle, three shRNA sequences were designed by GeneChem Inc. (Shanghai, China), and shRNA with the best kinetic parameters were selected for subsequent experiments. The shRNA target sequence was as follows: Negative Control (NC): 5´-TTCTCCGAACGTGTCACGT-3´; VHL-LV-94: 5´-CAGGTCGCTCTATGAAGACTT-3´; VHL-LV-95: 5´-GTGCCATCCCTCAATGTTGAT-3´; VHL-LV-96: 5´-CTGCCTTTGTGGCTCAACTTT-3´. The lentiviral vector system consisted of three plasmids: GV112, pHelper 1.0, and pHelper 2.0 vectors (provided by GeneChem Inc.). Among them, the clone sites of GV112 were AgeI and EcoRI. Then the NC and three target sequences were inserted into the GV112 vector by double enzyme digestion and T4 DNA ligase, and the recombinant plasmid was extracted and verified by Sanger sequencing. All enzymes were purchased from Thermo Fisher Scientific. Next, the recombinant GV112, pHelper 1.0, and pHelper 2.0 were co-transfected into 293T cells for lentivirus packaging (45). The lentiviral liquids were collected, and the quality was tested for later use.

The sensitivity of different cells to lentivirus was different, so pre-experiment should be carried out before transfection to determine the multiplicity of infection (MOI = (Virus titer × Virus volume)/cell number). The PC12 cells were seeded in a six-well plate and cultured until 50% confluence the day before transfection. On the next day, when the cells were cultured to the appropriate density, the transfection experiment was carried out. Then, 1.5 µg/ml puromycin (Sigma, USA) was used to select stable expression clones, and the knockdown efficiency was verified by RT-qPCR and Western blotting. The cell line with the best silencing effect was used in subsequent experiments.

### Quantitative Real-Time PCR

After grinding the frozen clinical tissue with liquid nitrogen (the cell line goes directly to the next step), RNA was extracted by the TRIzol method, and then the mRNA was reverse transcribed into cDNA (Thermo Scientific, USA) using the RevertAid First Strand cDNA Synthesis Kit (#K1622). Then, the Bio-Rad CFX96 real-time PCR system (Bio-Rad, CA, USA) was used to perform SYBR Green real-time quantitative PCR. The relative expression of mRNA transcripts in carcinoma and para-cancerous tissues was determined by SYBR® premix Ex Taq II Kit (Takara, Beijing, China) and target gene specific primers were listed in **Table S1**. The relative expression of different target genes was analyzed by Livak method (46).

### Western Blotting Assay

The total cell protein was lysed and extracted with RIPA buffer (PMSF, Solarbio, China) containing 1 mm of phenylmethanesulfonyl fluoride, and then quantified using the BCA protein assay kit (Beyotime, Shanghai, China). Subsequently, the equal molecular proteins from the lysates were separated using 12% SDS-PAGE gel and transferred to the polyvinylidene fluoride membranes (Millipore Corp, Billerica, MA, USA). After blocking with 5% skimmed milk in Tris buffer for 2 h at room temperature, the membranes were incubated in the target primary antibody at 4°C overnight (ABclonal Technology, Wuhan, China). After incubating the secondary antibody, the protein level was detected by the enhanced ECL luminescence reagent on a chemiluminescence imager (SmartChemi420, Sage Creation Science Co. Ltd., Beijing).

### Protein Extraction and Trypsin Digestion

Three parallel groups of negative control cells and LV-95 cells were sent for mass spectrometry detection. By counting with a hemocytometer, the number of cells in each dish was about 3~5 × 10^5^/ml. The samples were homogenized three times in the ice-cold lysis buffer (8 M urea, 1% Protease Inhibitor Cocktail) using an Ultrasonic Cell Disruptor (Scientz). After centrifugation at 4°C for 10 min, the cell debris was removed, and the cell supernatant was collected and the protein concentration was determined with the BCA kit according to the instructions (Beyotime Biotechnology, China). For digestion, the protein solution was reduced with 5 mM dithiothreitol for 30 min at 56°C and alkylated with 11 mM iodoacetamide in the dark at room temperature for 15 min. The protein samples were diluted by adding 100 mM tetraethyl ammonium bromide (TEAB) to urea concentration of less than 2 M. Finally, trypsin was added at a mass ratio of trypsin to protein of 1:50, and the first digestion was carried out overnight, and then at a ratio of trypsin to protein of 1:100 for the second digestion for 4 h.

After trypsinization, the peptides were desalted using a Strata XC18 SPE column (Phenomenex) and vacuum dried. Peptides were reconstituted in 0.5 M TEAB and processed according to the manufacturer’s protocol for TMT kit (Thermo Scientific, USA). The tryptic peptides were fractionated into different components by high pH reverse-phase HPLC using Thermo Betasil C18 column (5 μm particles, 10 mm ID, 250 mm length).

### LC-MS/MS Analysis and Database Search

The peptides were dissolved in the mobile phase A [0.1% (v/v) formic acid aqueous solution] of liquid chromatography, and then separated using the nanoElute ultra-high-performance liquid chromatography (UHPLC) system. Mobile phase A was an aqueous solution containing 0.1% formic acid, and mobile phase B was an acetonitrile solution containing 0.1% formic acid. The peptides were processed from NSI sources, and then tandem mass spectrometry (MS/MS) was performed in Q ExactiveTM Plus (Thermo) connected to online UHPLC. The applied electrospray voltage was 2.0 kV. The m/z scan range was 350 to 1,800 for full scan, and intact peptides were detected in the Orbitrap with a resolution of 70,000. Then used the NCE to set 28 to the selected peptides for MS/MS and detected these fragments in Orbitrap with a resolution of 17,500. A data-dependent procedure alternated between one MS scan and the subsequent 20 MS/MS scans with an interval of 15.0 s. The automatic gain control (AGC) was set to 5E4, and fixed first mass was set as 100 m/z.

### Database Search

We used Maxquant search engine (v.1.5.2.8) to process the obtained MS/MS data. Tandem mass spectra were searched against human UniProt database concatenated with reverse decoy database. Trypsin/P was designated as a lyase, allowing up to four missing cleavages. In the first search, the mass tolerance of precursor ions was set to 20 ppm, and 5 ppm in the Main search, and the mass tolerance of fragment ions was set to 0.02 Da. The carbamoyl group on Cys was designated as a fixed modification, and acetylation modification and oxidation on Met were specified as variable modifications. FDR was adjusted to <1% and the lowest score for modified peptides was set >40. Subsequently, the data searched in the database were respectively annotated with GO, domain, KEGG Pathway, and Subcellular Localization.

### Gene Ontology, Kyoto Encyclopedia of Genes and Genomes, and Protein Domain Enrichment Analysis

Gene Ontology (GO) annotation was derived from the UniProt-GOA database (http://www.ebi.ac.uk/GOA/), and GO enrichment analysis was performed with InterProScan platform (v.5.14-53.0 http://www.ebi.ac.uk/interpro/). Firstly, the identified protein ID was converted into UniProt ID, and then mapped to GO IDs through protein ID. If the UniProt-GOA database didn’t annotate some identified proteins, based on the protein sequence alignment method, InterProScan software was used to annotate the GO function of the protein. Secondly, GO annotations of proteins were divided into three categories: biological processes, cell composition, and molecular functions. Moreover, the KAAS server (v.2.0 http://www.genome.jp/kaas-bin/kaas_main) was used to perform the “Kyoto Encyclopedia of Genes and Genomes” (KEGG) annotation, and the KEGG database was used for pathway enrichment analysis. Furthermore, The InterPro database (http://www.ebi.ac.uk/interpro/) was used to analyze the enrichment of differentially expressed protein functional domains. Finally, the Fischer exact double-ended test method was used to test the differentially expressed proteins in the background of the identified protein, and the enrichment test with a corrected *P-value* of less than 0.05 was considered statistically significant.

### Enrichment-Based Clustering

Further hierarchical clustering was performed based on the functional classification of differentially expressed proteins. Firstly, we collated all the categories and their *P* value obtained after enrichment, and then filtered for those categories that are enriched in at least one of the clusters with a *P* value <0.05. Then the filtered *P* value matrix was transformed by the function x = −log10 (*P-value*). Finally, for each functional category, these x values were converted to z. Then, these z-scores were clustered by one-way hierarchical clustering (Euclidean distance, average linkage clustering) in Genesis. The cluster membership was visualized by using the heat map of “Heatmap. 2” function in the “gplots” R-package.

The *P* value was calculated by two-sample two-tailed t-test method. In terms of specification, the modulus (VHL shRNA/WT) where the differential expression change exceeds 1.3 was used as the change threshold for significant up or down regulation. To study the relationship between different proteins, all differentially expressed proteins (Fold change >1.3 or <0.769, VHL shRNA/WT) were searched against the STRING database version 11 to understand the protein-protein interactions. The protein-protein interaction network chart was plotted according to the following rules: only the interactions between proteins belonging to the search data set are selected to exclude external candidates; the STRING defined a metric called “confidence score,” which was used to define the confidence of the interaction, and we selected the confidence score > 0.7 (high confidence) for all interactions; the confidence score of the protein was higher, indicating that the protein were more important in the network. In addition, we labeled the different functions and types of proteins to distinguish the different functions of different proteins.

### Scratch and Transwell Assays

The cells were plated in 6 cm petri dish with 1 × 10^6^ cells per well. Then scratched each petri dish with a 200 µl pipette tip and Images were taken with inverted microscope on the same day and for the next 2 days. The Image J software developed by the National Institutes of Health (NIH) was used to analyze the changes in the scratched area.

In the migration test, 5 × 10^5^ cells were seeded in serum-free media in the upper well of an 8 µm pore transwell (BD), and the medium containing 20% FBS was placed at the bottom of the well. The cells were fixed with 4% paraformaldehyde and stained with crystal violet after 48 h and five random 10× images were taken from each well for quantification.

### Sanger Sequencing and Bioinformatics Prediction

The DNA of the samples was detected by Sanger sequencing to determine the mutation sites. Then the Protein Variation Effect Analyzer (PROVEAN: http://provean.jcvi.org/seq_submit.php) online prediction software was used to filter sequence variants to identify non-synonymous or indel variants that were predicted to be functionally important.

### Statistical Analysis

All experiments were verified more than three times for biological replicates and each subgroup was also repeated at least three times. The Graphpad prism 8.0 software (GraphPad Software Inc., San Diego, CA, USA) was used for variance analysis. All data were expressed as mean ± standard error (SEM).The one-way ANOVA with a *post-hoc* test method (Student-Newman-Keuls test) were used to analyze the differences between the mean values. The probability value of less than 0.05 was considered statistically significant.

## Results

### VHL Mutations in PPGL and Construction of VHL-KD PC12 Cell Line

**Figure 1:**
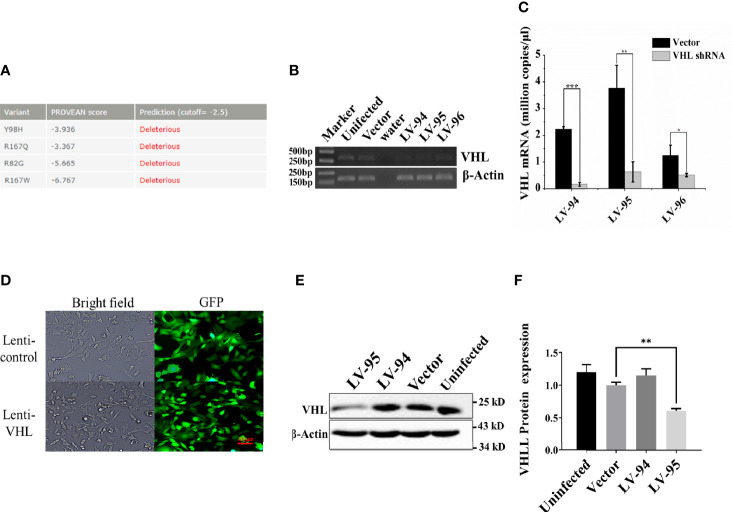
Constructing a VHL-KD PC12 cell line using shRNA. **(A)** The Protein Variation Effect Analyzer (PROVEAN) was used to filter sequence variants to identify non-synonymous or indel variants. The default threshold was −2.5. The variants with a score equal to or below −2.5 were considered “deleterious,” and the variants with a score above −2.5 were considered “neutral.” **(B)** We detected the expression of VHL mRNA in PC12 cells by qRT-PCR. At a specific number of cycles, the reaction was stopped, and the PCR product was used to run agarose gel electrophoresis to observe the mRNA relative expression. **(C)** Compared with the empty vector-transfected group, qRT-PCR verified the expression of VHL mRNA in VHL-KD and empty vector-transfected group, and each group was subjected to three biological replicates. The bar performed by Mean ± SEM; *P < 0.05, **P < 0.01, ***P < 0.001, n = 3. **(D)** After transfection, the GV248 recombinant vector could be stably expressed in PC12 cells. All un-transfected cells were cleared by puromycin [ρ (Puro) = 1.5 μg/ml, MOI = 12.5]. **(E)** Western blot was used to detect the expression of VHL protein in PC12 in the experimental groups. **(F)** The histogram obtained by gray analysis showed the relative change of proteins (mean ± SEM) n = 3, **P < 0.01.

Since VHL mutation was the most common type of mutation in PPGL genes, we collected four clinical samples with VHL mutations, and the specific clinical information and mutation sites were shown in **Table 1**. The PROVEAN was used to predict the hazard of the four mutations, and the predicted scores were shown in **Figure 1A**. To study the influence of VHL mutations on the development of pheochromocytoma, we constructed stable VHL-KD PC12 cell line model and evaluated the efficiency of VHL-KD at mRNA and protein levels. Firstly, to ensure the optimal growth state of cells during the VHL-KD process, we carried out preliminary experiments to optimize the transfection conditions. After transfection and puromycin screening, the VHL shRNA mediated by recombinant plasmid GV248 was stably expressed in the PC12 cells. The GV248 vector had the GFP fluorescent protein gene, which could monitor transfection efficiency of the recombinant vector in the PC12 cells (**Figure 1D**). The specific information of GV248 carrier could be obtained from **Table S2**. After transfection, the expression of VHL mRNA was significantly down-regulated in the three RNAi groups. However, the interference efficiencies of three kinds of shRNA targeting three segments of VHL mRNA were different, and the interference efficiencies of LV-94 and LV-95 were more significant (**Figures 1B, C****)**. To further verify the efficiency of RNAi at protein level, western blot confirmed that only LV-95 shRNA could successfully inhibit the expression of pVHL (**Figures 1E, F****)**.

**Table 1:** Characteristics and mutation sites of four patients with VHL-mutated pheochromocytomas.

Serial No.	Gender	Age	Diagnosis & location	Other disease	Blood pressure	Immunohistochemical index	Mutant gene	Mutation site
1	Female	61	PCC (Right)	Sudden blindness	156/76	CgA (++), Syn (+), S-100 (+), SF-1 (−), Ki67 (<1%+), inhibin (−), Melan-A (−), P53 (−)	VHL	NM_000551.3: c.292T>C, p.Y98H
2	Male	32	PCC (Double)	Hepatic nodules	140/87	CgA++, Syn+, S-100−, SF-1, Ki67 < 1%, inhibin−, Melan-A−, P53−	VHL	NM_000551.3:c.500G>A, p.R167Q
3	Female	51	PCC (Left)	PNET*	141/84	CgA+++, Syn++, S-100+, SF-1+, Ki67 < 1%+, inhibin−, Melan-A−, P53−	VHL	NM_000551.3:c.244C>G, p.R82G
4	Male	49	PCC (Double)	None	129/88	CgA (++), Syn (++), S-100 (++), Ki67 (2%+), inhibin (+), Melan-A (−), P53 (−)	VHL	NM_000551.3:c.499C>T, p.R167W

*PNET, Primitive Neuroectodermal Tumor.

### pVHL Regulating Multiple Cell Functions

**Figure 2:**
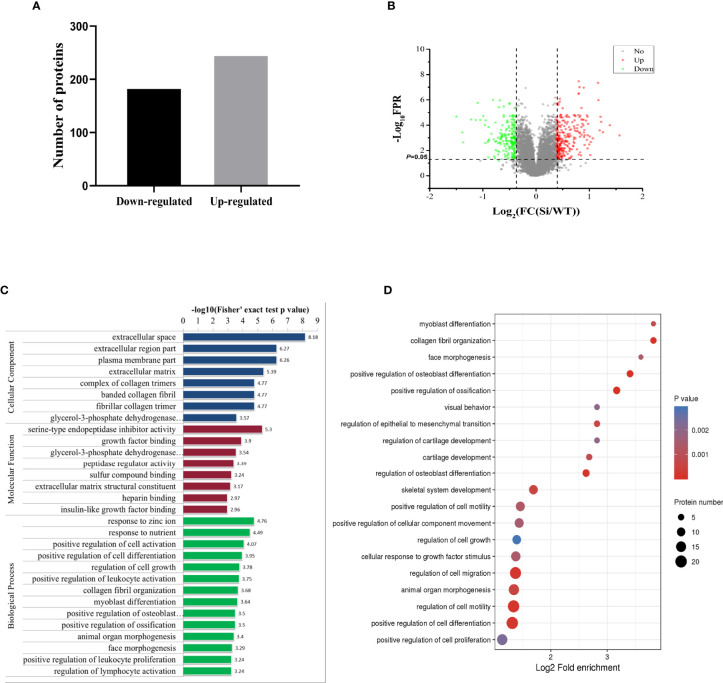
Proteomics analysis of VHL-KD PC12 cell line. **(A)** A bar graph showed the differential protein analyzed by TMT labeling quantitatively. **(B)** The volcano map of differentially expressed proteins. The abscissa denotes the ratios of differential expression proteins in the VHL-KD PC12 cell line *vs* those in the empty vector-transfected cell line; the ordinate represents the *P-value* between the two groups. **(C)** Differential protein was analyzed by GO function enrichment, and GO annotations were divided into three categories: Biological Process, Cellular Component, and Molecular Function. **(D)** The three main categories of differential proteins obtained by GO classification were further functionally analyzed. The circle in the figure indicated the number of differential proteins. The shade of the circle color represented the size of the *P* value. The darker the color of the circle represented the smaller the *P-value*; the lighter the color of the circle represented the larger the *P-value*.

To explore the biological processes regulated by pVHL, we carried out mass spectrometry analysis. We identified a total of 5,819 quantifiable proteins through the TMT-labeled proteomics quantitative platform. Among them, compared with the VHL-WT group, 248 proteins in the VHL-KD group were up-regulated, while 186 proteins were down-regulated (*P* < 0.05, **Figure 2A**, **Table S3**). These proteins were depicted in the quantitative volcanic distribution of differential proteins (**Figure 2B**, Fold Change >1.3 or <0.769, *P* < 0.05). Moreover, GO analysis annotated these proteins were divided into three major categories: biological processes, cell composition, and molecular functions, demonstrating the biological effects of proteins from different perspectives. Among them, the proteins related to cell proliferation, cell growth, and extracellular matrix were significantly enriched in these three functional categories (**Figure 2C**). Meanwhile, our results showed that the genes regulating cell growth, proliferation, differentiation, cell viability, and response to nutrients were significantly different in the VHL-KD group, and the functional enrichment analysis of the up-regulated proteins also showed that the enriched proteins were mainly involved in cell growth, proliferation, differentiation, and migration (**Figure 2D**).

**Figure 3:**
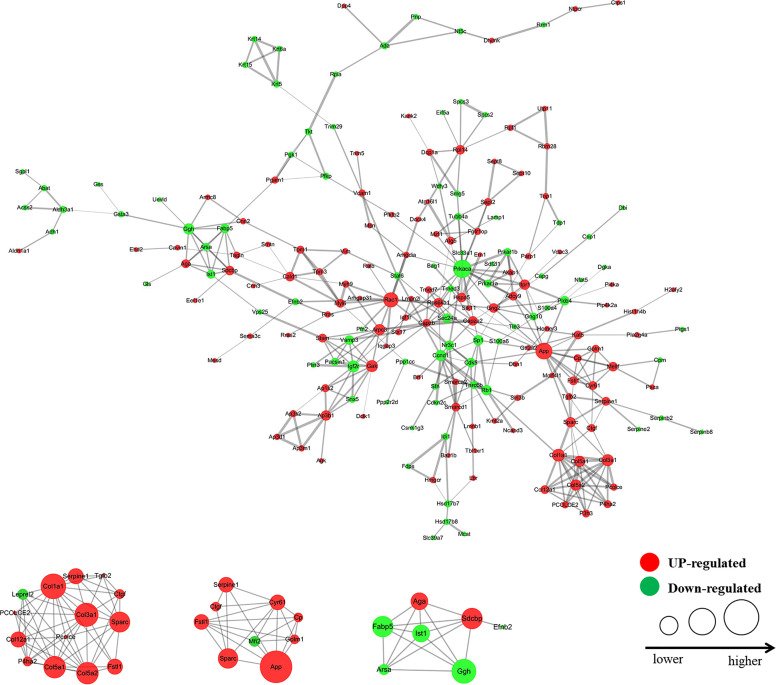
Interaction network diagram of the protein with fold change >1.3 (VHL shRNA/WT). The interactive network of 83 proteins and their differential expression in the VHL-KD cells *vs* those in the empty vector-transfected cells.

A total of 405 differentially expressed proteins were searched and included in the interaction network. Protein interaction analysis showed that most of the proteins related to proliferation, migration, and development in the VHL-KD group were significantly increased, but phosphorylation, ubiquitination, and ribosome-related proteins were significantly reduced in the VHL-KD group. Meanwhile, the up-regulations of cell surface receptor proteins, neurite outgrowth proteins, and axonogenesis proteins might promote synapseogenesis and enhance signal transmission and communication between cells (**Figure 3**).

### The Seven Novel Genes Up-Expressed in Pheochromocytoma Tumor

**Figure 4:**
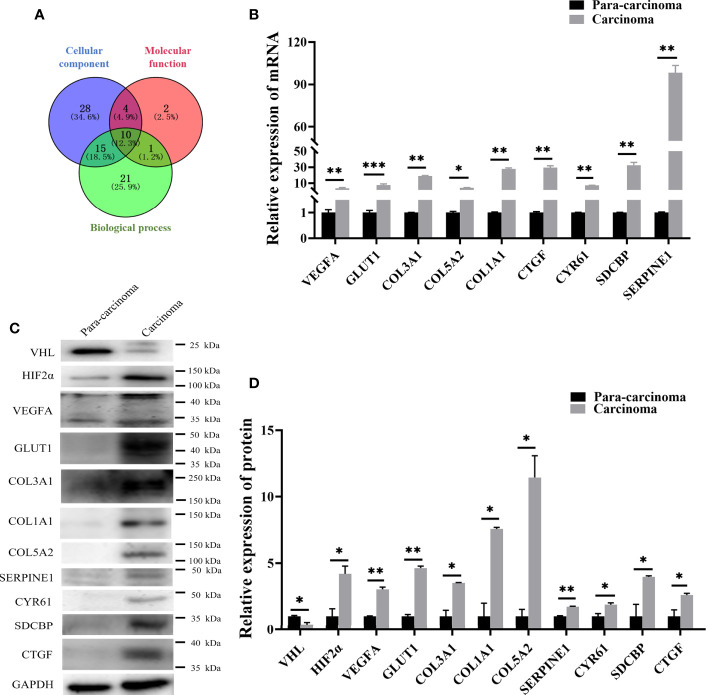
qRT-PCR and western blot confirmed that seven genes screened by proteomics analysis were also significantly up-regulated in pheochromocytoma tumors. **(A)** Venny diagram was used to screen the co-existing proteins among three groups of proteins. **(B)** qRT-PCR detected expression of seven genes in PCC carcinoma and para-carcinoma tissues. *significance between carcinoma *vs.* para-carcinoma group. *P < 0.05, **P < 0.01, ***P < 0.001, n = 4. **(C)** Western blot was used to detect protein expression in clinical tissue samples. *P < 0.05, **P < 0.01, n = 4. **(D)** The histogram obtained by gray analysis showed the relative change of proteins (mean ± SEM), n = 3, *P < 0.05, **P < 0.01.

In order to study the specific mechanism of pVHL affecting cell biological functions, we first collected the functional classification information of all proteomes and the corresponding enrichment *P* value (Fisher’s exact test), and then screened the significantly enriched functional classification (*P-value <0.05*) in at least one protein group. For the *P-value* obtained by Fisher’s exact test, the bubble chart showed the functional classification of significant enrichment of differential proteins and the results were shown in **Figure 2D**. As shown in the figure, the differentially expressed proteins mainly tended to the types of cell proliferation and migration. Therefore, we aimed to investigate the differentially expressed proteins associated with cell proliferation and migration. For biological process (BP), we selected 47 proteins related to cell proliferation, migration, and growth from BP group; for molecular function (MF), we selected 17 proteins related to growth factor binding, insulin-like growth factor binding, and peptidase regulatory activity from MF group. Furthermore, we selected 57 proteins related to extracellular matrix and extracellular space from the CC group as cell components (CC) while the screening workflow was shown in **Table S4**. In order to find differentially expressed proteins with important regulatory functions, we used the Venny Diagram online software (Venny-v.2.0) to select 10 differentially expressed proteins in these three parts (MF, CC, and BP groups) (**Figure 4A**). In order to further prove the clinical significance and application value of these proteins, we collected the tumor tissues of four patients with VHL mutation pheochromocytoma to verify the expression of these genes *in vivo*. We found that seven genes, including connective tissue growth factor (CTGF), Syndecan Binding Protein (SDCBP), Cysteine Rich Protein 61 (CYR61/CCN1), Type III Collagen A 1 Chain (COL3A1), Type I Collagen A 1 Chain (COL1A1), Type V Collagen A 2 Chain (COL5a2), and Serpin Family E Member 1 (SERPINE1), were significantly up-regulated in these tumor samples, which proved that VHL mutation activated Expression of these genes in pheochromocytoma. (**Figure 4B**). The up-regulation of seven genes in tumor tissues indicated that they played an important role in tumorigenesis and development. Meanwhile, western blot further confirmed the conclusions obtained by qRT-PCR (**Figures 4C, D**). The information of the seven genes in the proteomics quantitative analysis was shown in **Table 2**. In order to explore whether the overexpression of these genes was related to hypoxia-inducible factor (HIF), we detected HIFα and its downstream proteins vascular endothelial growth factor A (VEGFA) and glucose transporter 1 (Glut1) in VHL mutated carcinoma and para-carcinoma tissues. We found that in tumor tissues, HIF2α accumulated significantly, while VEGF and Glut1 were significantly overexpressed at both the mRNA and protein levels (**Figures 4B–D**).

**Table 2:** Specific information on differential expression of seven proteins in proteomics.

Protein accession	Gene name	Ratio(shRNA/WT)	*q*-value*	Biological process	Cellular component	Molecular function
Q9R1E9	Connective Tissue Growth Factor (CTGF)	1.763	0.000422	regulation of cell death and growth; regulation of G0 to G1 transition; regulation of cell differentiation and motility.	extracellular matrix; intracellular membrane-bounded organelle	fibronectin binding; carbohydrate derivative binding; growth factor activity;
Q9JI92	Syndecan Binding Protein (SDCBP)	2.093	0.001103	regulation of vesicle-mediated transport; positive regulation of cell migration; positive regulation of cell proliferation;	extracellular exosome; nucleoplasm; nuclear outer membrane-endoplasmic reticulum membrane network;	growth factor binding; cell adhesion molecule binding;
Q66HT5	Cysteine Rich Protein 61 (CYR61)	2.286	0.000256	positive regulation of cell migration; angiogenesis; regulation of cell growth;	extracellular matrix;	insulin-like growth factor binding; integrin binding; cell adhesion molecule binding;
P13941	Collagen Alpha-1 (III) Chain (COL3A1)	2.313	2.17E-05	nervous system development; negative regulation of cell development; regulation of cell migration;	supramolecular complex; extracellular matrix component;	structural molecule activity; cell adhesion molecule binding; growth factor binding;
P02454	Collagen Alpha-1 (I) Chain (COL1A1)	1.832	3.66E-05	positive regulation of canonical Wnt signaling pathway response to transforming growth factor beta; positive regulation of epithelial to mesenchymal transition;	membrane-bounded organelle; secretory vesicle; extracellular matrix component;	growth factor binding; extracellular matrix structural constituent;
F1LQ00	Collagen Type V Alpha 2 Chain (COL5A2)	1.875	0.000243	negative regulation of cell differentiation; cellular response to oxygen-containing compound;	proteinaceous extracellular matrix; supramolecular complex;	structural molecule activity; SMAD binding;
F1LM16	Serpin Family E Member 1 (SERPINE1)	1.343	0.005075	positive regulation of cell migration; positive regulation of vasculature development positive regulation of chemotaxis;	extracellular exosome; extracellular vesicle	protease binding; peptidase inhibitor activity

*q-value indicate the p-value after FDR correction.

### HIF2α Regulated the Overexpression of these Seven Novel Genes

**Figure 5:**
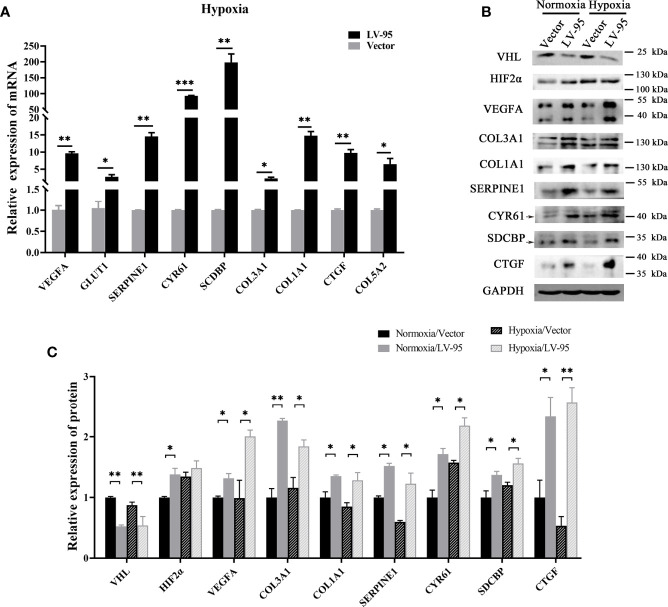
HIF-2α may mediate the overexpression of these seven genes. **(A)** The VHL-KD cell line (LV-95) and the empty vector-transfected cell line (Vector) were treated with hypoxia, and then qRT-PCR was used to detect the mRNA expression of seven target genes in the cells (mean ± SEM), n = 3, *P < 0.05, **P < 0.01, ***P < 0.001. **(B, C)** The western blot was used to detect the change of protein expression in cells when VHL is knocked down; the histogram indicated the multiple of protein changes (mean ± SEM), n = 3, **P < 0.01.

We further explored the mechanism of pVHL regulating these seven novel genes. Similar to other work, we found that HIF2α accumulated when VHL was knocked down (**Figure 5B**). In the case of abnormal accumulation of HIF2a, VEGF and Glut1 were significantly overexpressed (**Figures 5A, B**). To explore the role of HIF2α in the expression of these seven genes, we treated VHL-KD cells with hypoxia and observed the expression levels of these seven genes. After 24 h of hypoxia, the expression of six genes including CTGF, SDCBP, CCN1, COL3A1, COL1A1, and SERPINE1 significantly increased in the cells. Western blot showed no significant change of COL5a2 expression (**Figure 5B, C**) maybe due to its low expression in the cell. Therefore, HIF-2α might mediate the expression of these seven genes.

### VHL-KD Changes Cell Morphology and Promotes Cell Proliferation and Migration

**Figure 6:**
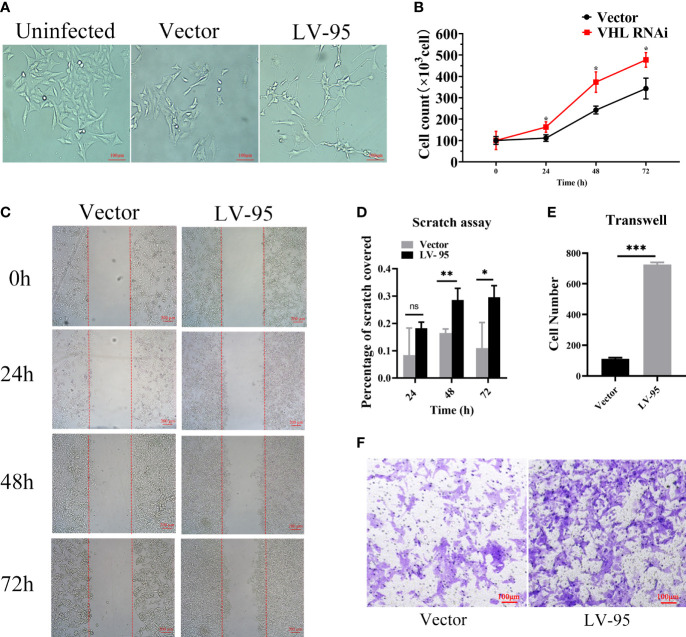
VHL-KD changed the cell morphology and promoted the proliferation and migration of PC12 cells. **(A)** Phase contrasted 10× images of VHL-KD (LV-95) and empty vector-transfected (Vector) PC12 cells. **(B)** Growth chart of VHL-KD and VHL empty vector-transfected cells, *P < 0.05. **(C, D)** Phase-contrast micrographs illustrating migration by wound healing of PC12 cells transduced with empty vector or VHL shRNA. Images were taken at different time points after wounding, as indicated. n = 3, ns: non significant, *P < 0.05, **P < 0.01. **(E, F)** Down: Micrographs illustrating trans-migration of PC12 cells transduced with empty vector or VHL shRNA. Up: Bar graphs indicated trans-migration relative to empty vector transduced cells. ***P < 0.001.

Since the discovered seven novel genes were related to cell proliferation and migration, we wanted to know whether VHL-KD would promote proliferation and migration. We observed that the morphology of VHL-KD cells changed, and the neurites of VHL-KD cells were longer than those of uninfected and empty vector transfected cells (**Figure 6A**). Furthermore, VHL-KD cells proliferated faster than empty vector-transfected cells (**Figure 6B**). Scratch and transwell assays showed that VHL-KD cells had stronger migration ability as compared to empty vector-transfected (**Figures 6C–F**).

## Discussion

As a rare neuroendocrine tumor, PPGL were mostly benign and could be cured by surgery (47). However, due to the limitations of precise diagnostic tools and effective treatment methods, metastatic PPGL had become a major challenge in the medical field (48, 49). Nowadays, more and more attention had been paid to the targeted therapy of PPGL (50). Therefore, optimizing the diagnostic efficiency of metastatic PPGL and finding more effective biological target molecules had gradually become the trend of clinical and basic research (51). In this study, the most relevant tumor malignant indicators and essential biological characteristics were used as screening criteria, and high-throughput technology was used to comprehensively evaluate the molecular mechanism of tumor pathogenesis. As we know, targeted therapy needs long-term and painstaking exploration to obtain more effective treatments (52).

As the key protein of VHL syndrome, pVHL regulated the expression of different tumor genes (53). The patients with VHL germline mutations were susceptible to VHL disease, which was an autosomal dominant syndrome (54). The multiple subtypes of VHL disease indicated that pVHL had multiple cellular functions (55). Other related studies also showed that VHL was involved in other functions besides the regulation mechanism of HIF1α-mediated proteasome degradation induced by hypoxia (19). Chitrakar et al. found that in Th17 cells, VHL was involved in the regulation of a variety of cellular pathways (56), including glycolytic pathways that were indirectly or directly inhibited by protein-coding genes (57). Furthermore, the VHL also controlled the function of innate immune cells (58), and the interleukin 33 receptor directly interacted with VHL (59). Our results suggested that pVHL was related to interleukin-related proteins. In order to study the role of pVHL in hypoxia induced pathway, we carried out the whole protein quantitative analysis. Our study showed that the differentially expressed proteins caused by VHL inactivation were mainly concentrated in clusters related to proliferation and migration. The deregulation of HIF2α could promote tumor development (60), but HIF2α had an undeveloped function that was largely independent of ARNT, which could affect gene transcription, cell differentiation, proliferation, and tumor metastasis and growth (61, 62). Sun et al. reported that VHL mutation promoted the proliferation, migration, and tumorigenesis of clear cell renal cell carcinoma (ccRCC) cells through the bridging function of SALL4 (63) and inhibited the senescence of ccRCC cells (64). Kondo et al. proved that pVHL achieved tumor suppression by inhibiting HIF2α (23). Our results conferred the seven downstream genes related to cell proliferation and migration regulated by the VHL/HIF2α axis.

From the complicated network controlled by pVHL, we found seven novel genes related to cell proliferation and migration. Firstly, our results showed that SERPINE1 expression was significantly up-regulated in the VHL-KD PC12 cell line, suggesting its distinctive biological significance. In a series of cell biology and molecular biology experiments, Yang et al. proved that overexpression of SERPINE1, which promoted the proliferation, invasion, and migration of ccRCC cells, was used as an independent prognostic factor for patients with gastric cancer (65). Secondly, our results showed that the expression of SDCBP was significantly increased *in vivo*. Related studies reported that the metastasis and spread of cancer cells were indirectly completed by many discrete processes, such as invasion, intravascular invasion, and angiogenesis (66), and SDCBP was a unique gene that promoted metastasis (67). Moreover, preclinical studies confirmed that inhibiting SDCBP from a genetic or pharmacological perspective could effectively inhibit cell metastasis (68). The expression of CTGF in our results was also significantly increased. Therefore, in our findings, the overexpression of SDCBP might promote the metastasis of pheochromocytoma. The low expression of CTGF was associated with the high overall survival rates of neuroblastoma patients, indicating the important role of CTGF in tumors (69). CTGF promoted the deposition of extracellular matrix and the proliferation of fibroblasts, thereby causing vascular diseases (70). Moreover, the phase-dependent regulation of CTGF was crucial for the malignancy of competent cancer cells (71). We discovered three collagen family proteins which were significantly up-regulated in PCC carcinoma. In liver cancer, there were relatively many studies on the function of COL1A1. Ma et al. proved that COL1A1, which promoted the HCC cell proliferation and invasion and the formation of tumor balls, was significantly up-regulated in liver cancer and enhanced carcinogenicity (72). Additionally, COL1A1 highly expressed in human breast and gastric cancer, while COL1A2 highly expressed in gastric cancer, and affected the prognosis (73, 74). As members of the collagen family, both COL3A1 and COL5A2 had significant value in the prognosis of gastric cancer (75). In our research results, they were significantly overexpressed in pheochromocytoma tissues and cell lines. Finally, the enrichment of CYR61 was very significant in VHL-KD cells. Mustafa Ilhan et al. demonstrated that overexpression of CYR61 promoted Notch1-induced migration, invasion, and anchorage-independent growth of the normal breast cancer cell line MCF10A, suggesting its importance in cancer progression (76). Thus, it could be concluded that the above seven genes regulated the proliferation, invasion, and metastasis of various tumors, suggesting that they could provide potential biomarkers and therapeutic targets for effective treatment of PPGL patients.

In conclusion, we found that the inactivation of pVHL in pheochromocytoma led to the up-regulation of the seven novel genes, such as CTGF, SDCBP, CYR61, COL3A1, COL1A1, COL5A2, and SERPINE1. The seven novel genes were closely related to cell proliferation, migration, and differentiation. Therefore, our results indicated that the seven proteins might serve as important diagnostic and therapeutic candidates for pheochromocytoma.

## Data Availability Statement

The original contributions presented in the study are publicly available, and the original data can be found on the Internet through the data set identifier of PXD021190: http://www.ebi.ac.uk/pride.

## Ethics Statement

The studies involving human participants were reviewed and approved by the Ethics Committee of the School of Life Sciences, Central South University. The patients/participants provided their written informed consent to participate in this study.

## Author Contributions

FZ and LL designed and supervised the experiments and modified and contributed towards writing the manuscript. SG performed the experiments and wrote the initial manuscript. ZL helped with the experiments and cell modeling. JS and YP collected clinical samples and helped with data. All authors contributed to the article and approved the submitted version.

## Funding

This work was supported by Natural Science Foundation of Hunan Province (No. 2018JJ6133), Innovation Driven Program of Central South University (No. 020CX046), Changsha Municipal Natural Science Foundation (No. kq2007062), and Founds for the Shenghua Yuying talents program of Central South University.

## Conflict of Interest

The authors declare that the research was conducted in the absence of any commercial or financial relationships that could be construed as a potential conflict of interest.
