# Supplementary material for: Seven Novel Genes Related to Cell Proliferation and Migration of VHL-Mutated Pheochromocytoma
Source: Front Endocrinol (Lausanne). 2021 Mar 22;12:598656. doi: 10.3389/fendo.2021.598656 (PMC8021008; doi:10.3389/fendo.2021.598656)
Supplement: Supplementary file 1 [file Table_1.docx]

| **Gene name** | **Primer type** | **Detailed sequence** | **Length** | **Product length (bp)** |
| --- | --- | --- | --- | --- |
| β-Actin | Forward primer | TGACGTGGACATCCGCAAAG | 20 | 205 |
|  | Reverse primer | CTGGAAGGTGGACAGCGAGG | 20 |  |
| CTGF | Forward primer | GGTGTGGCTTTAGGAGCAGT | 20 | 177 |
|  | Reverse primer | TGATGGCTGGAGAATGCACA | 20 |  |
| SDCBP | Forward primer | CTTGTGGGCTAGAATCCTGCAA | 22 | 148 |
|  | Reverse primer | CGTGAGGGATAGGAGCAGAAG | 21 |  |
| CYR61/CCN1 | Forward primer | CCGAGGTGGAGTTGACGAGA | 20 | 85 |
|  | Reverse primer | TCCATTCCAAAAACAGGGAGC | 21 |  |
| COL3A1 | Forward primer | CCTGAAGCTGATGGGGTCAA | 20 | 151 |
|  | Reverse primer | TAGTCTCACAGCCTTGCGTG | 20 |  |
| COL1A1 | Forward primer | AATGTGGTTCGTGACCGTGA | 20 | 181 |
|  | Reverse primer | AGCCTTGGTTGGGGTCAATC | 20 |  |
| COL5A2 | Forward primer | GATGGCAAACTGGGCGGAAG | 20 | 178 |
|  | Reverse primer | CTGACAAGGGGCAGGTTTCC | 20 |  |
| SERPINE1 | Forward primer | AGAACCTGGGAATGACCGAC | 20 | 169 |
|  | Reverse primer | ATGCGGGCTGAGACTATGAC | 20 |  |

**Table S1 Specific Sequences of Primers Used for Quantitative Real-Time PCR Assay of Human Tumors.**
