# Supplementary material for: Seven Novel Genes Related to Cell Proliferation and Migration of VHL-Mutated Pheochromocytoma
Source: Front Endocrinol (Lausanne). 2021 Mar 22;12:598656. doi: 10.3389/fendo.2021.598656 (PMC8021008; doi:10.3389/fendo.2021.598656)
Supplement: Supplementary file 2 [file Table_2.doc]

**Table S2 Lentiviral Vector Information of GV248**

| Vector name | GV248 | | | |
| --- | --- | --- | --- | --- |
| General | 11468bp | 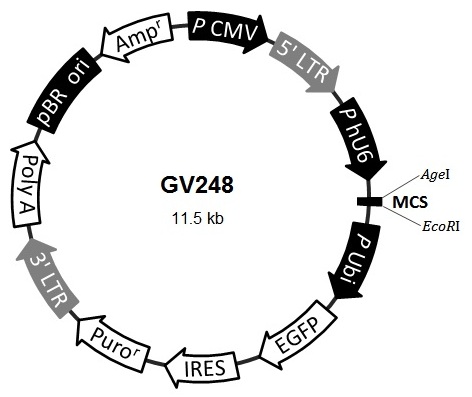 | | |
| psi | 1067-1204 |
| RRE | 1680-1913 |
| ORF frame 1 | 1558-2445 |
| hU6Promoter | 2602-2869 |
| EGFP | 4216-4935 |
| Puromycin | 5536-6135 |
| IRES | 4945-5529 |
| AmpR | 11309-10449 |
| CAG_enhance | 318-605 |
| Control insert sequence | TTCTCCGAACGTGTCACGT |
| Component order | hU6-MCS-Ubiquitin-EGFP-IRES-puromycin | |  | |
| Primer locations and sequences | H1-F(2479-2502)GGAAAGAATAGTAGACATAATAGC | | | Ubi-R(3136-3115)：ATGTCCTTCTGCTGATACTGGG |

Note: The manual can be obtained through：http://www.genechem.com.cn/service/index.php?ac=gene&at=vector_search&keyword=GV248

IRES: Internal ribosome entry site; RRE: Rev Response element; ORF: Open-reading frame; EGFP: enhanced Green Fluorescent Protein; AmpR: Ampicillin resistance gene.
