# Supplementary material for: Seven Novel Genes Related to Cell Proliferation and Migration of VHL-Mutated Pheochromocytoma
Source: Front Endocrinol (Lausanne). 2021 Mar 22;12:598656. doi: 10.3389/fendo.2021.598656 (PMC8021008; doi:10.3389/fendo.2021.598656)
Supplement: Supplementary file 3 [file Table_3.docx]

**Table S3 Specific Information for All Proteins with Absolute Fold change >1.3 (VHL shRNA/WT)**

| **Protein accession No. in Uniprot** | **Gene name** | **Ratio** | **Regulated** | ***P*-value** | **Protein description** |
| --- | --- | --- | --- | --- | --- |
| F1LVM4 | Ap3s2 | 2.962 | Up | 0.00064 | AP complex subunit sigma |
| A0A0G2K2L1 | Podxl | 2.611 | Up | 0.000102 | Podocalyxin |
| Q9Z1H9 | Cavin3 | 2.387 | Up | 0.000363 | Caveolae-associated protein 3 |
| P36860 | Ralb | 2.333 | Up | 5.52E-05 | Ras-related protein Ral-B |
| P13941 | Col3a1 | 2.313 | Up | 2.17E-05 | Collagen alpha-1(III) chain |
| Q66HT5 | Cyr61 | 2.286 | Up | 0.000256 | Cysteine rich protein 61 |
| P31232 | Tagln | 2.25 | Up | 1.07E-06 | Transgelin |
| A0A1W2Q6E9 | Msn | 2.242 | Up | 4.57E-08 | Moesin |
| Q9JI92 | Sdcbp | 2.093 | Up | 0.001103 | Syntenin-1 |
| Q62908 | Csrp2 | 2.04 | Up | 0.002158 | Cysteine and glycine-rich protein 2 |
| D3ZGL3 | Hhip | 2.028 | Up | 0.02332 | Hedgehog-interacting protein |
| A0A0G2K4R9 | Eogt | 2.012 | Up | 0.000799 | EGF domain-specific O-linked N-acetylglucosamine transferase |
| M0RBJ0 | Gng2 | 1.999 | Up | 0.003436 | Guanine nucleotide-binding protein subunit gamma |
| D3ZKW7 | Fam57a | 1.972 | Up | 7.83E-05 | "Family with sequence similarity 57, member A |
| F1LQZ0 | Tmem65 | 1.961 | Up | 0.000616 | Transmembrane protein 65 |
| D3ZPQ3 | Xaf1 | 1.958 | Up | 0.006924 | XIAP-associated factor 1 |
| B5DFK6 | Ap3d1 | 1.953 | Up | 2.16E-05 | Adaptor-related protein complex 3 subunit delta 1 |
| D4AEL2 | Golm1 | 1.941 | Up | 0.001478 | Golgi membrane protein 1 |
| A0A0G2JU83 | Lbr | 1.939 | Up | 1.67E-05 | Lamin-B receptor |
| F1M7X5 | Dpp4 | 1.92 | Up | 4.32E-05 | Dipeptidyl peptidase 4 |
| B0BNM8 | Cinp | 1.911 | Up | 0.098037 | "RCG38713, isoform CRA_b |
| Q9R0I8 | Pip4k2a | 1.883 | Up | 0.003705 | Phosphatidylinositol 5-phosphate 4-kinase type-2 alpha |
| Q6IRG9 | Ap3m1 | 1.881 | Up | 0.000623 | AP-3 complex subunit mu-1 |
| F1LQ00 | Col5a2 | 1.875 | Up | 0.000243 | Collagen type V alpha 2 chain |
| Q6DGG9 | Camlg | 1.87 | Up | 0.000857 | Calcium signal-modulating cyclophilin ligand |
| B1WBV2 | Sfrp2 | 1.864 | Up | 0.000378 | Secreted frizzled-related protein 2 |
| G3V6P7 | Myh9 | 1.861 | Up | 1.1E-07 | "Myosin, heavy polypeptide 9, non-muscle |
| Q5U2R7 | Mesd | 1.861 | Up | 3.78E-05 | LRP chaperone MESD |
| Q6AY90 | Fam192a | 1.851 | Up | 0.000104 | "Family with sequence similarity 192, member A |
| P02454 | Col1a1 | 1.832 | Up | 3.66E-05 | Collagen alpha-1(I) chain |
| G3V7V3 | Slc27a4 | 1.83 | Up | 7.85E-05 | "Solute carrier family 27 (Fatty acid transporter), member 4 |
| F1LTX4 | Lrrn4 | 1.809 | Up | 0.00058 | Leucine-rich repeat neuronal 4 |
| B1WC02 | Ctps1 | 1.806 | Up | 4.34E-06 | CTP synthase |
| B2RYN3 | Eef1e1 | 1.805 | Up | 0.000143 | Eukaryotic translation elongation factor 1 epsilon 1 |
| G3V8L9 | Cavin1 | 1.804 | Up | 3.64E-05 | Caveolae-associated protein 1 |
| Q5XI69 | Dhx40 | 1.797 | Up | 0.024245 | Probable ATP-dependent RNA helicase DHX40 |
| F1LVT1 | Zfp827 | 1.791 | Up | 0.172622 | IgLON family member 5 |
| Q5XI73 | Arhgdia | 1.78 | Up | 1.77E-05 | Rho GDP-dissociation inhibitor 1 |
| D3ZBS9 | Smarcd1 | 1.775 | Up | 0.014156 | "SWI/SNF related, matrix associated, actin dependent regulator of chromatin, subfamily d, member 1 (Predicted) |
| P05712 | Rab2a | 1.775 | Up | 0.000821 | Ras-related protein Rab-2A |
| Q9Z1Z9 | Pdlim7 | 1.765 | Up | 0.000382 | PDZ and LIM domain protein 7 |
| Q9R1E9 | Ctgf | 1.763 | Up | 0.000422 | Connective tissue growth factor |
| D3ZD89 | Naa15 | 1.761 | Up | 9.92E-08 | "N(alpha)-acetyltransferase 15, NatA auxiliary subunit |
| P23928 | Cryab | 1.76 | Up | 2.31E-05 | Alpha-crystallin B chain |
| M0R3V4 | Mydgf | 1.744 | Up | 3.44E-08 | Myeloid-derived growth factor |
| P13832 | Rlc-a | 1.744 | Up | 3.14E-07 | Myosin regulatory light chain RLC-A |
| D4ADK7 | Meltf | 1.742 | Up | 0.021396 | Antigen p97 (Melanoma associated) identified by monoclonal antibodies 133.2 and 96.5 (Predicted) |
| Q4V893 | Gpalpp1 | 1.742 | Up | 3.19E-07 | GPALPP motifs-containing protein 1 |
| P35571 | Gpd2 | 1.734 | Up | 1.58E-05 | "Glycerol-3-phosphate dehydrogenase, mitochondrial |
| G3V6M4 | Capn6 | 1.724 | Up | 0.0003 | Calpain-6 |
| D4AE80 | Dcp1a | 1.718 | Up | 9.69E-05 | Decapping mRNA 1A |
| Q7TT49 | Cdc42bpb | 1.706 | Up | 0.000176 | Serine/threonine-protein kinase MRCK beta |
| Q6IRI7 | Mafk | 1.704 | Up | 0.008257 | MAF bZIP transcription factor K |
| F7F3Z1 | Lman2l | 1.693 | Up | 0.000304 | "Lectin, mannose-binding 2-like |
| F1LSW7 | Rpl14 | 1.671 | Up | 1.96E-05 | 60S ribosomal protein L14 |
| Q99PD6 | Tgfb1i1 | 1.668 | Up | 0.001096 | Transforming growth factor beta-1-induced transcript 1 protein |
| D3ZCS4 | Iqgap3 | 1.659 | Up | 0.000265 | IQ motif-containing GTPase-activating protein 3 |
| G3V640 | Timm44 | 1.656 | Up | 1.66E-05 | Mitochondrial import inner membrane translocase subunit TIM44 |
| P29534 | Vcam1 | 1.65 | Up | 4.17E-05 | Vascular cell adhesion protein 1 |
| A0A0G2JWD6 | Ap3b1 | 1.648 | Up | 0.000175 | AP-3 complex subunit beta |
| M0R776 | Mrps36 | 1.645 | Up | 0.002563 | Mitochondrial ribosomal protein S36 |
| D4A0H1 | Reep3 | 1.639 | Up | 0.000924 | Receptor expression-enhancing protein |
| O35260 | Nacc1 | 1.639 | Up | 0.00012 | Nucleus accumbens-associated protein 1 |
| Q9QX80 | Hnrnpab | 1.637 | Up | 1.62E-05 | CArG-binding factor A |
| D3ZF45 | Larp4b | 1.634 | Up | 0.00134 | "La ribonucleoprotein domain family, member 4B |
| O88884 | Akap1 | 1.632 | Up | 0.002164 | "A-kinase anchor protein 1, mitochondrial |
| C9DRP5 | Pfkfb2 | 1.629 | Up | 0.004655 | "6-phosphofructo-2-kinase/fructose-2, 6-bisphosphatase 2 variant 4 |
| D3ZUP8 | Lmbrd2 | 1.619 | Up | 0.004339 | LMBR1 domain-containing 2 |
| D3ZKW6 | Upk3b | 1.604 | Up | 5.85E-05 | Uroplakin 3B |
| Q5FVH0 | C1qtnf5 | 1.595 | Up | 0.008296 | Complement C1q tumor necrosis factor-related protein 5 |
| D4ADG3 | Clk1 | 1.585 | Up | 0.029458 | CDC-like kinase 1 |
| D3ZNF4 | Tbl1xr1 | 1.577 | Up | 0.000216 | Transducin (Beta)-like 1X-linked receptor 1 (Predicted) |
| B5DF66 | Supt7l | 1.574 | Up | 0.03736 | SPT7-like STAGA complex gamma subunit |
| D3ZHD6 | Rbm15b | 1.57 | Up | 0.129023 | Ubiquitin protein ligase E3C |
| D3ZN05 | --- | 1.569 | Up | 0.000702 | Uncharacterized protein |
| F1MA20 | Rpf1 | 1.562 | Up | 0.007942 | Ribosome production factor 1 |
| G3V7H6 | Arg2 | 1.555 | Up | 0.000243 | Arginase |
| D4A987 | Arhgap31 | 1.552 | Up | 0.043456 | Cdc42 GTPase-activating protein (Predicted) |
| O08662 | Pi4ka | 1.546 | Up | 0.000484 | Phosphatidylinositol 4-kinase alpha |
| D4A3K3 | Dlg5 | 1.544 | Up | 0.006902 | Discs large MAGUK scaffold protein 5 |
| O08628 | Pcolce | 1.544 | Up | 0.011302 | Procollagen C-endopeptidase enhancer 1 |
| Q4KM58 | Ctnnbip1 | 1.539 | Up | 0.000879 | "Catenin, beta-interacting protein 1 |
| E9PTY0 | Lrrcc1 | 1.538 | Up | 0.000145 | Leucine-rich repeat and coiled-coil centrosomal protein 1 |
| F1M2K6 | Pkp4 | 1.537 | Up | 0.040463 | Plakophilin 4 |
| P18437 | Hmgn2 | 1.537 | Up | 2.25E-05 | Non-histone chromosomal protein HMG-17 |
| M0RD26 | PCOLCE2 | 1.536 | Up | 0.002864 | Procollagen C-endopeptidase enhancer 2 |
| B0BNJ0 | Sin3b | 1.532 | Up | 0.00072 | "RCG38713, isoform CRA_b |
| A0A0G2K4K3 | Abcc3 | 1.524 | Up | 0.35044 | Interferon gamma-induced GTPase |
| D4A542 | Gprasp2 | 1.513 | Up | 0.004883 | G protein-coupled receptor-associated sorting protein 2 |
| A0A0G2JSR9 | Sdf4 | 1.508 | Up | 5.58E-05 | 45 kDa calcium-binding protein |
| A0A0G2K535 | Baz2b | 1.508 | Up | 0.018776 | "Bromodomain adjacent to zinc finger domain, 2B |
| M0R8A4 | --- | 1.508 | Up | 0.000643 | Uncharacterized protein |
| D3Z9L0 | Agk | 1.506 | Up | 0.000498 | Acylglycerol kinase |
| P06687 | Atp1a3 | 1.502 | Up | 0.00188 | Sodium/potassium-transporting ATPase subunit alpha-3 |
| G3V9E3 | Cald1 | 1.501 | Up | 0.000841 | "Caldesmon 1, isoform CRA_b |
| D3ZQ09 | Zcchc24 | 1.5 | Up | 0.003964 | Zinc finger CCHC-type-containing 24 |
| A0A1B0GWQ5 | Elavl2 | 1.499 | Up | 2.21E-05 | ELAV-like protein |
| P25113 | Pgam1 | 1.498 | Up | 1.55E-05 | Phosphoglycerate mutase 1 |
| Q642A8 | Ubr7 | 1.497 | Up | 0.002105 | Similar to chromosome 14 open reading frame 130 |
| A0A0G2K6J5 | Myl6 | 1.491 | Up | 7.91E-05 | Myosin light polypeptide 6 |
| F1M0V0 | Zfp280d | 1.49 | Up | 0.055 | UEV and lactate/malate dehyrogenase domains |
| D3ZSV1 | Ift20 | 1.478 | Up | 0.23428 | Calponin (Fragment) |
| G3V9Z6 | Sept8 | 1.476 | Up | 2E-05 | Septin 8 (Predicted) |
| Q6T5F2 | Ugt1a2 | 1.475 | Up | 0.012964 | UDP-glucuronosyltransferase |
| D4AA11 | Med21 | 1.474 | Up | 0.05298 | RCG36668 |
| F1LYJ8 | Gramd4 | 1.474 | Up | 0.001721 | GRAM domain-containing 4 |
| A0A0H2UHB6 | Upk1b | 1.471 | Up | 0.001918 | RCG52686 |
| D3ZFK6 | Atg16l1 | 1.47 | Up | 0.000203 | Autophagy-related 16-like 1 |
| D3ZEU8 | Fam76a | 1.469 | Up | 0.001665 | "Family with sequence similarity 76, member A |
| F1M0L3 | Kmt2a | 1.468 | Up | 0.003539 | Histone-lysine N-methyltransferase |
| B1WC28 | H2afy2 | 1.465 | Up | 0.000944 | Core histone macro-H2A |
| D4A900 | Filip1l | 1.459 | Up | 5.91E-05 | Filamin A-interacting protein 1-like |
| P37397 | Cnn3 | 1.45 | Up | 0.000659 | Calponin-3 |
| A0A0H2UI02 | Stk11 | 1.449 | Up | 0.026659 | "Serine/threonine kinase 11 (Predicted), isoform CRA_a |
| P67999 | Rps6kb1 | 1.449 | Up | 0.006775 | Ribosomal protein S6 kinase beta-1 |
| Q99MH9 | Bbs2 | 1.449 | Up | 0.144404 | Attractin |
| D3ZRX9 | Cnn2 | 1.443 | Up | 0.000119 | Calponin (Fragment) |
| O08984 | Lbr | 1.442 | Up | 0.002077 | Lamin-B receptor |
| Q4V7C1 | Fgfr1op | 1.442 | Up | 0.024484 | FGFR1 oncogene partner |
| F1M943 | Armc8 | 1.441 | Up | 0.002884 | Armadillo repeat-containing 8 |
| Q3T1K5 | Capza2 | 1.44 | Up | 0.006898 | F-actin-capping protein subunit alpha-2 |
| F1LMD9 | Gak | 1.439 | Up | 0.000397 | Cyclin-G-associated kinase |
| G3V8R1 | Nucb2 | 1.439 | Up | 4.19E-05 | "Nucleobindin 2, isoform CRA_b |
| O88498 | Bcl2l11 | 1.439 | Up | 0.157159 | 2'-deoxynucleoside 5'-phosphate N-hydrolase 1 |
| Q6P796 | Spint2 | 1.433 | Up | 0.010558 | "Serine peptidase inhibitor, Kunitz type, 2 |
| Q6P9V1 | Cd81 | 1.427 | Up | 0.017498 | Tetraspanin |
| M0R5U4 | Adcy9 | 1.423 | Up | 0.009602 | Adenylate cyclase 9 |
| Q99MY2 | Nudt4 | 1.421 | Up | 0.012799 | Diphosphoinositol polyphosphate phosphohydrolase 2 |
| D4ABA5 | Smtn | 1.419 | Up | 0.001663 | "RCG35999, isoform CRA_a |
| B0BN99 | Hmgb3 | 1.416 | Up | 4.4E-05 | Hmgb3 protein |
| Q6IRF5 | Pla2g4a | 1.415 | Up | 0.000378 | Phospholipase A2 |
| A0A0G2KAJ7 | Col12a1 | 1.413 | Up | 0.000363 | Collagen alpha-1(XII) chain |
| A0A0H2UHR7 | Flnc | 1.413 | Up | 4.64E-06 | Filamin-C |
| Q6P6G9 | Hnrnpa1 | 1.41 | Up | 2.5E-05 | Heterogeneous nuclear ribonucleoprotein A1 |
| B4F7E7 | Mpp5 | 1.405 | Up | 0.030875 | Membrane palmitoylated protein 5 |
| D3ZVT3 | Card6 | 1.405 | Up | 0.001264 | "Caspase recruitment domain family, member 6 |
| G3V665 | Castor2 | 1.405 | Up | 0.23792 | Bromodomain adjacent to zinc finger domain protein 1B |
| F7FLS6 | Lzts2 | 1.402 | Up | 0.060817 | Semaphorin 3C |
| A0A0G2K5F4 | Sept10 | 1.401 | Up | 0.001877 | Septin-10 |
| A0A0G2KA75 | Kat5 | 1.401 | Up | 0.043219 | Histone acetyltransferase |
| D4A053 | Shroom2 | 1.4 | Up | 0.006258 | Protein Shroom2 |
| Q5FWT5 | Qrsl1 | 1.4 | Up | 0.026024 | "Glutamyl-tRNA(Gln) amidotransferase subunit A, mitochondrial |
| P30919 | Aga | 1.397 | Up | 0.006676 | N(4)-(Beta-N-acetylglucosaminyl)-L-asparaginase |
| Q3MHT4 | Tut1 | 1.397 | Up | 0.053641 | Guanine nucleotide-binding protein subunit gamma |
| Q66H19 | Srfbp1 | 1.397 | Up | 0.010636 | Serum response factor-binding protein 1 |
| Q66H54 | Fam160a2 | 1.397 | Up | 0.083596 | Serum response factor-binding protein 1 |
| F1LNF3 | Nfxl1 | 1.394 | Up | 0.02744 | "Nuclear transcription factor, X-box-binding-like 1 |
| M0R4L9 | Wdcp | 1.394 | Up | 0.008458 | Uncharacterized protein |
| A0A0G2JXX9 | Ap1s2 | 1.393 | Up | 0.002522 | AP complex subunit sigma |
| D3ZTX0 | Tmed7 | 1.393 | Up | 9.62E-05 | Transmembrane emp24 domain-containing protein 7 |
| B5DF55 | Stam | 1.392 | Up | 2.31E-05 | RCG55706 |
| A0A0G2K471 | Igtp | 1.391 | Up | 0.002585 | Interferon gamma-induced GTPase |
| G3V9N1 | Pgam5 | 1.391 | Up | 0.000244 | RCG21137 |
| A0A0A0MY31 | Itpr1 | 1.39 | Up | 0.002063 | "Inositol 1,4,5-trisphosphate receptor type 1 |
| B5DFB0 | P3h3 | 1.39 | Up | 0.033019 | "Leprecan-like 2 (Predicted), isoform CRA_b |
| A0A0G2JTJ5 | Ctdspl2 | 1.389 | Up | 0.0624 | 45 kDa calcium-binding protein |
| B1WBW7 | Traf7 | 1.388 | Up | 0.062841 | Secreted frizzled-related protein 2 |
| Q6AYG7 | Nfatc2ip | 1.388 | Up | 0.22502 | RCG43931 |
| D3ZBQ5 | Scarf2 | 1.387 | Up | 0.010062 | "Scavenger receptor class F, member 2 |
| Q6Q0N3 | Nt5dc2 | 1.387 | Up | 1.53E-05 | 5'-nucleotidase domain-containing protein 2 |
| Q5U306 | Akap8l | 1.382 | Up | 0.017319 | A kinase (PRKA) anchor protein 8-like |
| A0A0G2JSR0 | Vdac3 | 1.38 | Up | 2.48E-05 | Voltage-dependent anion-selective channel protein 3 |
| F1LPK1 | Il6st | 1.38 | Up | 0.001121 | Interleukin-6 receptor subunit beta |
| F1LSY7 | Ern1 | 1.379 | Up | 0.031602 | Endoplasmic reticulum to nucleus-signaling 1 |
| A0A0G2JYL4 | P4ha2 | 1.377 | Up | 1.51E-05 | Prolyl 4-hydroxylase subunit alpha 2 |
| A0A140TAF0 | Tpm3 | 1.375 | Up | 0.000138 | "RCG62531, isoform CRA_g |
| D3ZF21 | Gprin3 | 1.374 | Up | 0.007705 | GPRIN family member 3 |
| Q6AYF8 | Serpinb9 | 1.373 | Up | 0.004041 | RCG43931 |
| F7FHT4 | Sema3c | 1.369 | Up | 0.007336 | Semaphorin 3C |
| M0R3N4 | Vat1l | 1.367 | Up | 0.003158 | Vesicle amine transport 1-like |
| B2GV73 | Arpc3 | 1.366 | Up | 1.88E-05 | Actin-related protein 2/3 complex subunit 3 |
| D3ZIF0 | Zfp512 | 1.365 | Up | 0.003877 | Zinc finger protein 512 |
| D4A478 | Ntpcr | 1.365 | Up | 0.003175 | "Nucleoside-triphosphatase, cancer-related |
| D4A5K7 | Rbm28 | 1.365 | Up | 0.001364 | RNA-binding motif protein 28 |
| Q6P6Q5 | App | 1.365 | Up | 0.000396 | Amyloid-beta A4 protein |
| D4A456 | Lrch2 | 1.364 | Up | 0.006216 | Leucine-rich repeats and calponin homology domain-containing 2 |
| A0JPQ5 | RGD1560212 | 1.362 | Up | 1.38E-06 | "Similar to DNA segment, Chr 18, Wayne State University 98, expressed (Predicted), isoform CRA_c |
| O55004 | Rnase4 | 1.362 | Up | 0.21388 | 2'-deoxynucleoside 5'-phosphate N-hydrolase 1 |
| P31000 | Vim | 1.361 | Up | 8.09E-07 | Vimentin |
| Q2YDV1 | Dusp9 | 1.361 | Up | 0.001122 | Dual specificity protein phosphatase |
| D3ZRM5 | Rab23 | 1.36 | Up | 0.000681 | "RAB23, member RAS oncogene family |
| D4ABK7 | Hnrnph3 | 1.36 | Up | 0.021885 | "Heterogeneous nuclear ribonucleoprotein H3 (2H9) (Predicted), isoform CRA_c |
| Q4V7D8 | --- | 1.36 | Up | 0.008877 | UPF0711 protein C18orf21 homolog |
| Q91Y81 | Sept2 | 1.36 | Up | 6.04E-05 | Septin-2 |
| A0A0G2K3V7 | Top1 | 1.358 | Up | 0.00012 | DNA topoisomerase 1 |
| D3ZC46 | Tcf25 | 1.356 | Up | 0.018562 | Transcription factor 25 |
| D4A0Y0 | Trim5 | 1.356 | Up | 0.003164 | Tripartite motif-containing 5 |
| B1H261 | Tysnd1 | 1.354 | Up | 0.23298 | RAB6-interacting golgin |
| D4A8W8 | Brf1 | 1.354 | Up | 0.001619 | "BRF1 homolog, subunit of RNA polymerase III transcription initiation factor IIIB (S. cerevisiae) (Predicted), isoform CRA_a |
| Q9R1Q2 | Ccnl1 | 1.354 | Up | 0.40032 | Sodium bicarbonate cotransporter 3 |
| D3ZR79 | Tmem245 | 1.353 | Up | 0.049116 | Transmembrane protein 245 |
| A0A0A0MY46 | Ralgapa1 | 1.351 | Up | 0.010037 | Ral GTPase-activating protein subunit alpha-1 |
| D4A4N1 | Mzt1 | 1.351 | Up | 0.004603 | Mitotic spindle organizing protein 1 |
| Q3MQ06 | Atg5 | 1.351 | Up | 0.013764 | Autophagy protein 5 |
| D4A634 | Ranbp6 | 1.35 | Up | 0.018557 | RAN-binding protein 6 |
| D3ZAQ0 | Fundc2 | 1.349 | Up | 0.012204 | FUN14 domain-containing 2 |
| M0R4K3 | Zcchc14 | 1.347 | Up | 0.041443 | Zinc finger CCHC-type-containing 14 |
| M0R6X6 | --- | 1.347 | Up | 0.001177 | Uncharacterized protein |
| A0A096MJ03 | Asb12 | 1.346 | Up | 0.042645 | Ankyrin repeat and SOCS box-containing 12 |
| A0A0G2JVI5 | Afap1 | 1.346 | Up | 0.000902 | Actin filament-associated protein 1 |
| D3ZPM7 | Adam19 | 1.344 | Up | 0.010497 | ADAM metallopeptidase domain 19 |
| Q7TPB4 | Cd276 | 1.344 | Up | 0.007324 | CD276 antigen |
| D4A7W1 | Abhd2 | 1.343 | Up | 0.022222 | Abhydrolase domain containing 2 (Predicted) |
| F1LM16 | Serpine1 | 1.343 | Up | 0.005075 | Plasminogen activator inhibitor 1 |
| F1LYZ8 | Ppp1r21 | 1.343 | Up | 0.009677 | "Protein phosphatase 1, regulatory subunit 21 |
| P14841 | Cst3 | 1.343 | Up | 0.072321 | Lysosome-associated membrane glycoprotein 1 |
| F1M471 | Epm2aip1 | 1.342 | Up | 0.0015 | EPM2A-interacting protein 1 |
| F8WG88 | Fstl1 | 1.342 | Up | 0.000735 | Follistatin-related protein 1 |
| Q6RUV5 | Rac1 | 1.341 | Up | 5.76E-05 | Ras-related C3 botulinum toxin substrate 1 |
| Q9Z158 | Stx17 | 1.341 | Up | 0.008261 | Syntaxin-17 |
| M0R6T4 | Mms19 | 1.34 | Up | 0.00802 | "MMS19 homolog, cytosolic iron-sulfur assembly component |
| A0A0H2UHP1 | Aldh1a1 | 1.338 | Up | 0.031517 | Retinal dehydrogenase 1 |
| D3ZP87 | Fam92a | 1.338 | Up | 0.22892 | Transducin (Beta)-like 1X-linked receptor 1 (Predicted) |
| A0A0G2JTA7 | Rasal2 | 1.337 | Up | 0.082843 | 45 kDa calcium-binding protein |
| A0A0G2JUS0 | LOC108348175 | 1.337 | Up | 0.000101 | Protein quaking-like |
| D4A2C4 | Morc2 | 1.337 | Up | 0.001165 | MORC family CW-type zinc finger 2 |
| D4ACC2 | Kank2 | 1.336 | Up | 0.001097 | KN motif and ankyrin repeat domains 2 |
| F1LTJ5 | --- | 1.336 | Up | 0.001477 | Uncharacterized protein |
| P06761 | Hspa5 | 1.336 | Up | 2.51E-06 | Endoplasmic reticulum chaperone BiP |
| G3V8G5 | Glg1 | 1.335 | Up | 1.87E-06 | Golgi apparatus protein 1 |
| F1LWM1 | Ssh1 | 1.334 | Up | 0.000638 | Slingshot protein phosphatase 1 |
| P51639 | Hmgcr | 1.334 | Up | 0.035058 | 3-hydroxy-3-methylglutaryl-coenzyme A reductase |
| D3ZKI6 | Ralgapa2 | 1.333 | Up | 0.043398 | Ral GTPase-activating protein subunit alpha-2 |
| D3ZPE6 | Mrpl51 | 1.332 | Up | 0.11632 | Transducin (Beta)-like 1X-linked receptor 1 (Predicted) |
| D4A4H5 | Sdf2 | 1.332 | Up | 0.013778 | "Stromal cell derived factor 2 (Predicted), isoform CRA_b |
| Q6IE50 | Smoc1 | 1.332 | Up | 0.000596 | SPARC-related modular calcium binding protein 1 |
| Q9JMA8 | Extl3 | 1.332 | Up | 0.001725 | "Exostoses (Multiple)-like 3, isoform CRA_a |
| A0A0G2K2H0 | Axl | 1.331 | Up | 0.20422 | NLR family member X1 |
| D4A6I7 | Psca | 1.331 | Up | 0.027876 | Prostate stem cell antigen |
| P36201 | Crip2 | 1.331 | Up | 0.00036 | Cysteine-rich protein 2 |
| A0A0G2JXD9 | Dop1b | 1.33 | Up | 0.006858 | Dopey family member 2 |
| P62804 | Hist1h4b | 1.33 | Up | 0.000124 | Histone H4 |
| A0A0G2KB74 | Dip2a | 1.328 | Up | 0.147418 | Transmembrane protein 189 |
| D4A1K4 | Misp3 | 1.328 | Up | 0.134502 | Tripartite motif-containing 5 |
| M0R6K4 | Dock4 | 1.327 | Up | 0.001304 | Dedicator of cytokinesis 4 |
| A0A0G2JWB6 | Pxdn | 1.326 | Up | 0.006099 | Peroxidasin |
| O08950 | Gtf2a2 | 1.326 | Up | 0.002082 | Transcription initiation factor IIA subunit 2 |
| F1M265 | --- | 1.324 | Up | 0.000241 | Uncharacterized protein |
| D3ZEV8 | Susd2 | 1.323 | Up | 0.004535 | "Sushi domain containing 2 (Predicted), isoform CRA_a |
| Q5U2Q4 | Parp16 | 1.323 | Up | 0.153438 | NEDD8-conjugating enzyme UBE2F |
| P16975 | Sparc | 1.322 | Up | 0.003043 | SPARC |
| A0A1W2Q6Q2 | Dclk1 | 1.321 | Up | 4.41E-05 | Serine/threonine-protein kinase DCLK1 |
| B5DF84 | Utp11 | 1.321 | Up | 0.034044 | U3 small nucleolar RNA-associated protein 11 |
| B5DFF2 | Rbpms2 | 1.321 | Up | 0.010599 | "RNA-binding protein, mRNA-processing factor 2 |
| A0A140TAB8 | Igf1r | 1.32 | Up | 0.012277 | Tyrosine-protein kinase receptor |
| Q6AY55 | Dcakd | 1.32 | Up | 0.000161 | Dephospho-CoA kinase domain-containing protein |
| D3ZUJ5 | Dtymk | 1.319 | Up | 8.19E-05 | Deoxythymidylate kinase |
| F1LR29 | Frmd6 | 1.319 | Up | 0.057761 | Transmembrane protein 65 |
| G3V8W2 | Cds2 | 1.318 | Up | 0.130936 | "Nucleobindin 2, isoform CRA_b |
| D3ZAN3 | Ganab | 1.317 | Up | 0.000184 | Alpha glucosidase 2 alpha neutral subunit (Predicted) |
| D3ZCP6 | Amer1 | 1.317 | Up | 0.20548 | Transcription factor 25 |
| A0A0G2K652 | Cul9 | 1.315 | Up | 0.118536 | Septin-10 |
| G3V7U4 | Lmnb1 | 1.315 | Up | 2.26E-05 | Lamin-B1 |
| Q765A7 | Pgap1 | 1.315 | Up | 0.001299 | GPI inositol-deacylase |
| D3ZU63 | Stard13 | 1.314 | Up | 0.097555 | Transmembrane emp24 domain-containing protein 7 |
| F1LVR0 | Iglon5 | 1.314 | Up | 0.005362 | IgLON family member 5 |
| I6L9G5 | Rcn3 | 1.314 | Up | 0.089315 | Septin 8 (Predicted) |
| Q6AYU1 | Morf4l1 | 1.314 | Up | 0.002944 | Mortality factor 4-like protein 1 |
| A0A140UHX3 | Ncapd3 | 1.313 | Up | 0.04776 | "Non-SMC condensin II complex, subunit D3 |
| A6N6J5 | Wdr35 | 1.313 | Up | 0.135742 | "Protein kinase, cAMP-dependent, catalytic, alpha |
| A0A0H2UHX1 | Rtn3 | 1.312 | Up | 7.62E-05 | Reticulon |
| Q9R066 | Cxadr | 1.312 | Up | 0.00776 | Coxsackievirus and adenovirus receptor homolog |
| Q62651 | Ech1 | 1.311 | Up | 0.0004 | "Delta(3,5)-Delta(2,4)-dienoyl-CoA isomerase, mitochondrial |
| Q6AXN8 | Zscan21 | 1.311 | Up | 0.000222 | Zinc finger and SCAN domain-containing 21 |
| Q99J86 | Atrn | 1.311 | Up | 0.010524 | Attractin |
| A0A0G2K508 | Rras2 | 1.31 | Up | 4.23E-05 | RAS-related 2 |
| G3V6B1 | Tgfb2 | 1.31 | Up | 0.023784 | Transforming growth factor beta-2 |
| Q9Z2X5 | Homer3 | 1.31 | Up | 0.000957 | Homer protein homolog 3 |
| A0A0H2UHL9 | Dbn1 | 1.309 | Up | 3.7E-05 | Drebrin |
| D3ZBL5 | Inpp4a | 1.309 | Up | 0.053135 | FUN14 domain-containing 2 |
| G3V661 | Baz1b | 1.309 | Up | 2.12E-06 | Bromodomain adjacent to zinc finger domain protein 1B |
| E9PTG1 | Smarca2 | 1.308 | Up | 0.003939 | "SWI/SNF-related, matrix-associated, actin-dependent regulator of chromatin, subfamily a, member 2 |
| D3Z8L7 | Rras | 1.307 | Up | 0.022102 | Ras-related protein R-Ras |
| Q9JM15 | Slc38a1 | 1.305 | Up | 0.001299 | Sodium-coupled neutral amino acid transporter 1 |
| B0BNM4 | RGD1308134 | 1.304 | Up | 0.085102 | "RCG38713, isoform CRA_b |
| G3V7K3 | Cp | 1.304 | Up | 0.001315 | Ceruloplasmin |
| Q9Z0V5 | Prdx4 | 1.304 | Up | 0.004541 | Peroxiredoxin-4 |
| A0A0G2JSQ4 | Tpm1 | 1.302 | Up | 0.019961 | "Tropomyosin 1, alpha, isoform CRA_p |
| D3Z9M5 | Fkbp7 | 1.302 | Up | 0.018084 | Peptidylprolyl isomerase |
| Q9JI03 | Col5a1 | 1.302 | Up | 0.001542 | Collagen alpha-1(V) chain |
| P27008 | Parp1 | 1.301 | Up | 0.000262 | Poly [ADP-ribose] polymerase 1 |
| Q4V8H8 | Ehd2 | 1.301 | Up | 0.028019 | EH domain-containing protein 2 |
| Q5U2Z8 | Elovl1 | 1.301 | Up | 0.000644 | Elongation of very long chain fatty acids protein 1 |
| Q5XI32 | Capzb | 1.301 | Up | 0.000575 | F-actin-capping protein subunit beta |
| G3V6P3 | Zhx2 | 1.3 | Up | 0.019645 | Calpain-6 |
| Q5PQV5 | Tpbg | 1.3 | Up | 0.035343 | Serine/threonine-protein kinase DCLK2 |
| B2B9A9 | Efnb2 | 0.769 | Down | 0.003136 | Ephrin B2 |
| D3ZWR1 | Nt5c | 0.769 | Down | 0.002319 | "5', 3'-nucleotidase, cytosolic |
| G3V943 | Dnaaf5 | 0.769 | Down | 0.001183 | "Dynein, axonemal, assembly factor 5 |
| D3ZGB1 | Nfat5 | 0.768 | Down | 0.036277 | Nuclear factor of activated T-cells 5 |
| Q5BJY6 | Amdhd2 | 0.767 | Down | 0.000423 | N-acetylglucosamine-6-phosphate deacetylase |
| A0JPN0 | Rad18 | 0.765 | Down | 0.000404 | RAD18 E3 ubiquitin protein ligase |
| D3ZLC3 | Itprid2 | 0.765 | Down | 0.001038 | Sperm specific antigen 2 |
| O35820 | Dnph1 | 0.764 | Down | 0.002636 | 2'-deoxynucleoside 5'-phosphate N-hydrolase 1 |
| P05942 | S100a4 | 0.764 | Down | 5.96E-05 | Protein S100-A4 |
| Q32KK2 | Arsa | 0.764 | Down | 0.001217 | Arylsulfatase A |
| Q6AYC4 | Capg | 0.764 | Down | 0.001258 | Macrophage-capping protein |
| A0A0G2JSQ0 | Ppp2r2d | 0.763 | Down | 0.001939 | Serine/threonine-protein phosphatase 2A 55 kDa regulatory subunit B |
| D3ZZ48 | Slc35a1 | 0.763 | Down | 0.039323 | "Solute carrier family 35 (CMP-sialic acid transporter), member 1 (Predicted) |
| F1M4A7 | Gse1 | 0.763 | Down | 0.125538 | EPM2A-interacting protein 1 |
| P63255 | Crip1 | 0.763 | Down | 0.000375 | Cysteine-rich protein 1 |
| D4A6E8 | RGD1560065 | 0.762 | Down | 0.036739 | Similar to RIKEN cDNA 2410004B18 |
| P16617 | Pgk1 | 0.762 | Down | 1.86E-05 | Phosphoglycerate kinase 1 |
| Q66HG4 | Galm | 0.762 | Down | 0.003001 | Aldose 1-epimerase |
| D3ZHV3 | Mt1m | 0.761 | Down | 0.001161 | Metallothionein |
| P46413 | Gss | 0.76 | Down | 0.000205 | Glutathione synthetase |
| P51556 | Dgka | 0.76 | Down | 0.001282 | Diacylglycerol kinase alpha |
| Q6AY25 | Tmed3 | 0.76 | Down | 9.91E-05 | Transmembrane emp24 domain-containing protein 3 |
| O35760 | Idi1 | 0.759 | Down | 0.015558 | Isopentenyl-diphosphate Delta-isomerase 1 |
| B4F7C2 | Tubb4a | 0.758 | Down | 0.016078 | Tubulin beta chain |
| G3V779 | Lad1 | 0.758 | Down | 0.006637 | Ladinin-1 |
| A0A0G2JSU4 | Ndrg2 | 0.757 | Down | 0.136296 | 45 kDa calcium-binding protein |
| A0A0G2K6G2 | Apmap | 0.757 | Down | 0.000843 | Adipocyte plasma membrane-associated protein |
| B2GV97 | Sepsecs | 0.757 | Down | 0.015179 | O-phosphoseryl-tRNA(Sec) selenium transferase |
| G3V7Z3 | Nol3 | 0.757 | Down | 0.59918 | "Solute carrier family 27 (Fatty acid transporter), member 4 |
| G3V7Z4 | Serpine2 | 0.757 | Down | 0.031701 | Glia-derived nexin |
| P11030 | Dbi | 0.757 | Down | 5.73E-05 | Acyl-CoA-binding protein |
| Q568Z6 | Ist1 | 0.757 | Down | 0.000684 | IST1 homolog |
| D3ZDB9 | Nmral1 | 0.756 | Down | 0.000141 | NmrA-like family domain-containing protein 1 |
| Q5M8C7 | Txndc9 | 0.756 | Down | 0.029663 | Thioredoxin domain containing 9 |
| D3ZV75 | Mfsd1 | 0.755 | Down | 0.021185 | Major facilitator superfamily domain-containing 1 |
| Q66HK3 | Ptgs1 | 0.755 | Down | 4.47E-06 | Prostaglandin G/H synthase 1 |
| A0A0G2K1Q1 | Nlrx1 | 0.754 | Down | 0.001036 | NLR family member X1 |
| B2RZA8 | Slc30a6 | 0.754 | Down | 0.162418 | Prolyl-tRNA synthetase-associated domain-containing 1 |
| Q9JIT3 | Tle3 | 0.754 | Down | 0.004244 | Transducin-like enhancer protein 3 |
| D3ZAA9 | Mpp2 | 0.753 | Down | 0.012141 | MAGUK p55 subfamily member 2 |
| P13264 | Gls | 0.752 | Down | 0.00108 | "Glutaminase kidney isoform, mitochondrial |
| P50137 | Tkt | 0.752 | Down | 0.000138 | Transketolase |
| A0A0G2JXN8 | Osbpl8 | 0.751 | Down | 0.002358 | Oxysterol-binding protein |
| D3ZZT6 | Tsen54 | 0.751 | Down | 0.040676 | "RCG32786, isoform CRA_b |
| D4A9Y0 | Sdf2l1 | 0.751 | Down | 0.002182 | RCG36668 |
| F1LRS8 | Cd2ap | 0.751 | Down | 9.99E-05 | CD2-associated protein |
| F1LUD3 | --- | 0.751 | Down | 3.99E-05 | Uncharacterized protein |
| A0A0G2JYA4 | LOC100362453 | 0.75 | Down | 0.25524 | AP complex subunit sigma |
| D3ZPF2 | Mcat | 0.75 | Down | 0.009856 | Malonyl-CoA-acyl carrier protein transacylase |
| D4A7L6 | Rpia | 0.75 | Down | 0.008656 | Ribose 5-phosphate isomerase A |
| F1LWK7 | --- | 0.75 | Down | 0.052423 | IgLON family member 5 |
| G3V684 | Med15 | 0.75 | Down | 0.060623 | Multidrug resistance-associated protein 5 |
| D3ZD11 | Spcs2 | 0.749 | Down | 0.000764 | Signal peptidase complex subunit 2 |
| D4A4W6 | Slirp | 0.749 | Down | 0.000939 | "RCG20695, isoform CRA_b |
| F1M7M4 | Bmp2k | 0.748 | Down | 0.068217 | Endoplasmic reticulum metallopeptidase 1 |
| P85973 | Pnp | 0.747 | Down | 0.000277 | Purine nucleoside phosphorylase |
| R9PXV5 | Rb1 | 0.747 | Down | 0.006115 | Retinoblastoma-associated protein |
| A0A0A0MY24 | Gzmbl3 | 0.746 | Down | 0.000857 | Granzyme B-like 3 |
| D4A9T2 | Twsg1 | 0.746 | Down | 0.137599 | Carboxypeptidase M |
| D3Z9D2 | Fyco1 | 0.745 | Down | 1.69E-05 | FYVE and coiled-coil domain containing 1 (Predicted) |
| Q4V8K5 | Brox | 0.745 | Down | 0.000922 | BRO1 domain-containing protein BROX |
| Q6MGB4 | Slc39a7 | 0.745 | Down | 0.028504 | "H2-K region expressed gene 4, rat orthologue |
| G3V676 | Abcc5 | 0.744 | Down | 0.003336 | Multidrug resistance-associated protein 5 |
| P09034 | Ass1 | 0.744 | Down | 3.64E-05 | Argininosuccinate synthase |
| A0A0G2K007 | Stk39 | 0.743 | Down | 0.089758 | Prolyl 4-hydroxylase subunit alpha 2 |
| A0A0G2K0S1 | Calcoco1 | 0.743 | Down | 2.39E-05 | Calcium-binding and coiled-coil domain-containing protein 1-like |
| Q6AYR4 | Tor2a | 0.743 | Down | 0.2589 | Peptidyl-prolyl cis-trans isomerase |
| P47860 | Pfkp | 0.742 | Down | 0.03448 | "ATP-dependent 6-phosphofructokinase, platelet type |
| D3Z9Z9 | Smarcad1 | 0.739 | Down | 0.003084 | SWI/SNF-related matrix-associated actin-dependent regulator of chromatin subfamily A containing DEAD/H box 1 |
| Q5XIQ6 | Trmt2a | 0.739 | Down | 0.019441 | "RCG36750, isoform CRA_a |
| Q6AYQ9 | Ppic | 0.739 | Down | 0.043936 | Peptidyl-prolyl cis-trans isomerase |
| Q01714 | Sp1 | 0.738 | Down | 0.000316 | Transcription factor Sp1 |
| Q8CHN6 | Sgpl1 | 0.738 | Down | 7.72E-05 | Sphingosine-1-phosphate lyase 1 |
| Q9JHX4 | Casp8 | 0.738 | Down | 0.0659 | Transforming growth factor beta-1-induced transcript 1 protein |
| Q9R1N3 | Slc4a7 | 0.738 | Down | 0.002665 | Sodium bicarbonate cotransporter 3 |
| P05964 | S100a6 | 0.737 | Down | 0.003415 | Protein S100-A6 |
| P12369 | Prkar2b | 0.737 | Down | 0.20456 | "Aldehyde dehydrogenase, dimeric NADP-preferring |
| Q5I0D7 | Pepd | 0.737 | Down | 9.76E-05 | Xaa-Pro dipeptidase |
| Q66HG6 | Ca5b | 0.737 | Down | 0.007518 | "Carbonic anhydrase 5B, mitochondrial |
| G3V9A3 | Sfn | 0.736 | Down | 2.04E-05 | RCG31390 |
| Q5BK16 | Trex1 | 0.736 | Down | 0.055123 | N-acetylglucosamine-6-phosphate deacetylase |
| P14562 | Lamp1 | 0.735 | Down | 1.63E-05 | Lysosome-associated membrane glycoprotein 1 |
| Q3KRE3 | Gng10 | 0.735 | Down | 0.004344 | Guanine nucleotide-binding protein subunit gamma |
| Q8K3F3 | Ppp1r14b | 0.735 | Down | 0.000984 | Protein phosphatase 1 regulatory subunit 14B |
| B2RYW9 | Fahd2 | 0.734 | Down | 0.023123 | Fumarylacetoacetate hydrolase domain-containing protein 2 |
| D3ZAP9 | Gpd1l | 0.734 | Down | 0.00052 | Glycerol-3-phosphate dehydrogenase [NAD(+)] |
| D3ZDU5 | Pfn2 | 0.734 | Down | 0.000178 | Profilin |
| G3V824 | Igf2r | 0.734 | Down | 0.002257 | Insulin-like growth factor 2 receptor |
| P39948 | Ccnd1 | 0.734 | Down | 0.000104 | G1/S-specific cyclin-D1 |
| E9PT74 | Zdhhc20 | 0.733 | Down | 0.065178 | Golgi membrane protein 1 |
| D3ZZ25 | Zfp280c | 0.732 | Down | 0.162835 | "NDUFA4, mitochondrial complex-associated-like 2 |
| F1M0M3 | Uevld | 0.732 | Down | 0.000535 | UEV and lactate/malate dehyrogenase domains |
| Q99P74 | Rab27b | 0.732 | Down | 0.030558 | Ras-related protein Rab-27B |
| D3ZHB7 | Ube3c | 0.731 | Down | 0.005905 | Ubiquitin protein ligase E3C |
| D3ZXX8 | Ndufa4l2 | 0.731 | Down | 0.000425 | "NDUFA4, mitochondrial complex-associated-like 2 |
| Q62867 | Ggh | 0.731 | Down | 0.002218 | Gamma-glutamyl hydrolase |
| A0A0A0MXY3 | Csnk1g3 | 0.73 | Down | 0.036696 | Casein kinase I isoform gamma-3 |
| F1LNK2 | Plcb4 | 0.729 | Down | 0.004623 | Phosphoinositide phospholipase C |
| F1LQU9 | Sgsm3 | 0.729 | Down | 0.000997 | Small G protein-signaling modulator 3 |
| M0RA08 | Plin3 | 0.729 | Down | 0.001617 | Perilipin |
| P81377 | Prkar1b | 0.729 | Down | 0.01574 | cAMP-dependent protein kinase type I-beta regulatory subunit |
| B1H267 | Snx5 | 0.728 | Down | 6.02E-05 | Sorting nexin-5 |
| D3ZZA8 | Sec24a | 0.728 | Down | 0.002125 | "SEC24 homolog A, COPII coat complex component |
| D4A4L1 | Sohlh1 | 0.728 | Down | 0.000196 | Spermatogenesis and oogenesis-specific basic helix-loop-helix 1 |
| Q5HZW5 | Cd320 | 0.728 | Down | 0.00174 | CD320 antigen |
| P05370 | G6pdx | 0.727 | Down | 3.56E-05 | Glucose-6-phosphate 1-dehydrogenase |
| M0R8J3 | Jmy | 0.726 | Down | 0.00116 | Junction-mediating and regulatory protein |
| A0A0G2K9B2 | Gpcpd1 | 0.724 | Down | 0.002802 | Glycerophosphocholine phosphodiesterase GPCPD1 |
| O35815 | Atxn3 | 0.724 | Down | 0.002402 | Ataxin-3 |
| Q5I0K5 | Abhd10 | 0.72 | Down | 0.000921 | "Mycophenolic acid acyl-glucuronide esterase, mitochondrial |
| D4A7Z8 | Wnk3 | 0.715 | Down | 0.006441 | RCG38922 |
| D4AAR7 | Ccdc136 | 0.715 | Down | 0.058324 | RCG36668 |
| P06536 | Nr3c1 | 0.715 | Down | 0.001944 | Glucocorticoid receptor |
| A0A0G2JXT3 | Fdps | 0.712 | Down | 0.005476 | Farnesyl pyrophosphate synthase |
| P11883 | Aldh3a1 | 0.712 | Down | 1.92E-05 | "Aldehyde dehydrogenase, dimeric NADP-preferring |
| G3V9U0 | Acss2 | 0.711 | Down | 0.000282 | Acyl-CoA synthetase short-chain family member 2 |
| P62634 | Cnbp | 0.709 | Down | 2.15E-06 | Cellular nucleic acid-binding protein |
| A0A0G2K542 | Ugp2 | 0.706 | Down | 1.83E-06 | UTP--glucose-1-phosphate uridylyltransferase |
| O08836 | Igbp1 | 0.704 | Down | 0.008001 | Immunoglobulin-binding protein 1 |
| Q03114 | Cdk5 | 0.703 | Down | 0.000536 | Cyclin-dependent-like kinase 5 |
| D3ZJ32 | Esyt2 | 0.702 | Down | 0.000163 | Extended synaptotagmin 2 |
| G3V8P7 | LOC108348044 | 0.702 | Down | 0.001782 | Ras and Rab interactor 1 |
| A0A0U1RRV5 | Cluh | 0.701 | Down | 0.000118 | Clustered mitochondria protein homolog |
| G3V887 | Tcirg1 | 0.701 | Down | 0.005182 | V-type proton ATPase subunit a |
| P63025 | Vamp3 | 0.701 | Down | 0.000561 | Vesicle-associated membrane protein 3 |
| Q5U203 | Ube2f | 0.698 | Down | 0.021584 | NEDD8-conjugating enzyme UBE2F |
| D3ZEJ9 | RGD1310429 | 0.697 | Down | 0.038879 | Similar to Protein Njmu-R1 |
| E9PU64 | Scin | 0.694 | Down | 5.5E-05 | Scinderin |
| Q80Z30 | Ppm1e | 0.694 | Down | 0.017105 | Protein phosphatase 1E |
| Q7TP44 | --- | 0.689 | Down | 0.046879 | Ab2-390 |
| Q4QQU9 | Ostm1 | 0.688 | Down | 0.00348 | Osteopetrosis-associated transmembrane protein 1 |
| A1L1K8 | Habp4 | 0.687 | Down | 0.002645 | Intracellular hyaluronan-binding protein 4 |
| P38656 | Ssb | 0.686 | Down | 4.31E-05 | Lupus La protein homolog |
| Q9JMI1 | Aacs | 0.684 | Down | 6.25E-05 | Acetoacetyl-CoA synthetase |
| A0A0G2JWR2 | Pacsin1 | 0.683 | Down | 0.025976 | Protein kinase C and casein kinase substrate in neurons protein 1 |
| D4ABW9 | Trim29 | 0.682 | Down | 0.003099 | "Tripartite motif protein 29 (Predicted), isoform CRA_b |
| M0R4P9 | Ube2h | 0.681 | Down | 0.0002 | Ubiquitin-conjugating enzyme E2H |
| M0R8C6 | Slc14a1 | 0.681 | Down | 0.000602 | Urea transporter |
| F1M5Q6 | Fbxo21 | 0.68 | Down | 0.087144 | EPM2A-interacting protein 1 |
| P04904 | Gsta3 | 0.679 | Down | 0.001743 | Glutathione S-transferase alpha-3 |
| D3ZF12 | Spcs3 | 0.677 | Down | 0.001165 | Signal peptidase complex subunit 3 |
| O35077 | Gpd1 | 0.677 | Down | 0.000963 | "Glycerol-3-phosphate dehydrogenase [NAD(+)], cytoplasmic |
| A0A0G2K2S2 | Slc2a1 | 0.676 | Down | 0.000257 | "Solute carrier family 2, facilitated glucose transporter member 1 |
| B2RZA5 | Prorsd1 | 0.675 | Down | 0.00166 | Prolyl-tRNA synthetase-associated domain-containing 1 |
| D3ZKP6 | Mlkl | 0.675 | Down | 0.01156 | Mixed lineage kinase domain-like pseudokinase |
| Q5MPA9 | Dclk2 | 0.675 | Down | 0.007242 | Serine/threonine-protein kinase DCLK2 |
| A0A0G2KB37 | Tmem189 | 0.669 | Down | 0.032296 | Transmembrane protein 189 |
| A0A0H2UI26 | Steap3 | 0.668 | Down | 0.000741 | Metalloreductase STEAP3 |
| A0A0H2UHC3 | Tdp1 | 0.667 | Down | 0.021262 | Tyrosyl-DNA phosphodiesterase 1 |
| B2RZ08 | Tmem263 | 0.666 | Down | 0.000504 | RGD1563325 protein |
| Q1KQ07 | Stat6 | 0.666 | Down | 0.004384 | Signal transducer and activator of transcription |
| A0A1B0GWW0 | Ube3c | 0.662 | Down | 0.000655 | Ubiquitin protein ligase E3C (Fragment) |
| D3ZIP8 | --- | 0.661 | Down | 0.002963 | Uncharacterized protein |
| Q6DGG1 | Abhd14b | 0.657 | Down | 2.15E-05 | Protein ABHD14B |
| Q920P6 | Ada | 0.656 | Down | 3.34E-06 | Adenosine deaminase |
| P09456 | Prkar1a | 0.655 | Down | 1.62E-05 | cAMP-dependent protein kinase type I-alpha regulatory subunit |
| P63088 | Ppp1cc | 0.653 | Down | 0.010396 | Serine/threonine-protein phosphatase PP1-gamma catalytic subunit |
| F1M9C0 | Mapkapk2 | 0.651 | Down | 0.001804 | Mitogen-activated protein kinase-activated protein kinase 2 |
| D4AB36 | NEWGENE_6497122 | 0.65 | Down | 0.131597 | RCG36668 |
| D3ZHR8 | Tyw3 | 0.649 | Down | 0.158759 | Ubiquitin protein ligase E3C |
| G3V6P4 | Phospho1 | 0.646 | Down | 0.000102 | "Phosphatase, orphan 1 (Predicted) |
| Q9JHY1 | F11r | 0.644 | Down | 0.000682 | Junctional adhesion molecule A |
| G3V6C1 | Serpinb5 | 0.643 | Down | 0.000902 | "RCG24055, isoform CRA_b |
| Q4G075 | Serpinb1a | 0.642 | Down | 0.000504 | Leukocyte elastase inhibitor A |
| A0A0G2KB65 | LOC103690164 | 0.639 | Down | 0.050595 | Transmembrane protein 189 |
| D4AE65 | Rrp7a | 0.638 | Down | 0.30282 | Mitogen-activated protein kinase kinase kinase 20 |
| P55053 | Fabp5 | 0.638 | Down | 0.000858 | Fatty acid-binding protein 5 |
| Q6AYY8 | Slc33a1 | 0.637 | Down | 0.000642 | Acetyl-coenzyme A transporter 1 |
| A0A0G2K628 | Ski | 0.634 | Down | 0.3039 | Septin-10 |
| F1M6W2 | Ermp1 | 0.634 | Down | 0.000238 | Endoplasmic reticulum metallopeptidase 1 |
| Q4FZU2 | Krt6a | 0.633 | Down | 0.00128 | "Keratin, type II cytoskeletal 6A |
| A0A0G2K9M4 | Wdfy3 | 0.631 | Down | 0.000963 | WD repeat and FYVE domain-containing 3 |
| B5DF45 | Traf6 | 0.631 | Down | 0.003197 | TNF receptor-associated factor 6 |
| A0A0G2K6R0 | Tnrc6b | 0.628 | Down | 0.00234 | Trinucleotide repeat-containing 6B |
| A0A0G2KAZ7 | Hnrnpdl | 0.627 | Down | 0.000421 | Heterogeneous nuclear ribonucleoprotein D-like |
| B4F7A3 | Lgalsl | 0.627 | Down | 0.001236 | Galectin |
| P06757 | Adh1 | 0.624 | Down | 1.09E-06 | Alcohol dehydrogenase 1 |
| Q5M949 | Nipsnap3b | 0.622 | Down | 0.002418 | Nipsnap homolog 3A (C. elegans) |
| D4AE17 | Map3k20 | 0.62 | Down | 0.012876 | Mitogen-activated protein kinase kinase kinase 20 |
| P53987 | Slc16a1 | 0.617 | Down | 3.81E-06 | Monocarboxylate transporter 1 |
| F7FFR1 | Rars2 | 0.614 | Down | 0.064525 | "Lectin, mannose-binding 2-like |
| P50554 | Abat | 0.61 | Down | 0.021101 | "4-aminobutyrate aminotransferase, mitochondrial |
| A0A096MJA9 | Asph | 0.609 | Down | 0.000995 | Aspartate-beta-hydroxylase |
| F1LRM5 | Bag1 | 0.609 | Down | 0.002677 | BAG family molecular chaperone regulator 1 |
| Q5M821 | Ppm1h | 0.607 | Down | 0.011162 | Protein phosphatase 1H |
| Q6IFV3 | Krt15 | 0.595 | Down | 0.001918 | "Keratin, type I cytoskeletal 15 |
| F1LNP8 | Nectin1 | 0.591 | Down | 0.009516 | Nectin cell adhesion molecule 1 |
| A0A0H2UHE2 | Il1rn | 0.589 | Down | 6.21E-05 | Interleukin-1 |
| M0R7I5 | --- | 0.588 | Down | 0.002025 | Uncharacterized protein |
| P0C0A1 | Vps25 | 0.584 | Down | 0.041456 | Vacuolar protein-sorting-associated protein 25 |
| Q3T1J1 | Eif5a | 0.581 | Down | 0.029783 | Eukaryotic translation initiation factor 5A-1 |
| D3ZWA1 | Mindy2 | 0.575 | Down | 0.001355 | MINDY lysine 48 deubiquitinase 2 |
| P04177 | Th | 0.571 | Down | 1E-06 | Tyrosine 3-monooxygenase |
| Q8R5G4 | Cdkn2c | 0.559 | Down | 0.000476 | Cyclin-dependent kinase inhibitor 2C |
| F8WFF9 | Lrrc57 | 0.541 | Down | 0.001437 | Leucine-rich repeat-containing protein 57 |
| A1L1M0 | Prkaca | 0.539 | Down | 0.000142 | "Protein kinase, cAMP-dependent, catalytic, alpha |
| Q63318 | LOC100911104 | 0.538 | Down | 0.001663 | Ly6-C antigen |
| E9PU03 | Smg5 | 0.536 | Down | 0.035743 | SMG5 nonsense mediated mRNA decay factor |
| A0A0G2K0T8 | Fam25a | 0.534 | Down | 0.002163 | "Family with sequence similarity 25, member A |
| B1H222 | Gorab | 0.518 | Down | 4.14E-05 | RAB6-interacting golgin |
| A0A0G2K6P1 | Hsd17b8 | 0.517 | Down | 0.002603 | Estradiol 17-beta-dehydrogenase 8 |
| A0A0H2UHB1 | Hsd17b7 | 0.514 | Down | 9.86E-05 | 3-keto-steroid reductase |
| Q5U2Q5 | Rrm1 | 0.502 | Down | 1.86E-05 | Ribonucleoside-diphosphate reductase |
| A0A0H2UHI3 | Calcr | 0.484 | Down | 0.064296 | Interleukin-1 |
| Q6P6Q2 | Krt5 | 0.484 | Down | 3.88E-05 | "Keratin, type II cytoskeletal 5 |
| Q6IFV1 | Krt14 | 0.468 | Down | 1.74E-06 | "Keratin, type I cytoskeletal 14 |
| G3V804 | Sfxn3 | 0.456 | Down | 3.93E-05 | Sideroflexin |
| A0A0G2K7H6 | Serpinb8 | 0.429 | Down | 3.61E-05 | Serpin family B member 8 |
| Q6AY63 | Nudt5 | 0.387 | Down | 0.002322 | ADP-sugar pyrophosphatase |
| P29524 | Serpinb2 | 0.383 | Down | 0.000378 | Plasminogen activator inhibitor 2 type A |
| D4A9Q5 | Cpm | 0.354 | Down | 2.08E-05 | Carboxypeptidase M |
