# Supplementary material for: Seven Novel Genes Related to Cell Proliferation and Migration of VHL-Mutated Pheochromocytoma
Source: Front Endocrinol (Lausanne). 2021 Mar 22;12:598656. doi: 10.3389/fendo.2021.598656 (PMC8021008; doi:10.3389/fendo.2021.598656)
Supplement: Supplementary file 4 [file Table_4.docx]

**Table S4 Technical route and the workflow of the**  **a nalysis**

Protein Extraction and Trypsin Digestion

LC-MS/MS Analysis

GO Annotation

Biological Process

Cellular Component

Molecular Function

Ten proteins


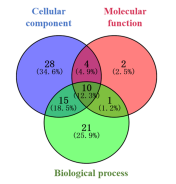


Associated with proliferation or migration
